# Global burden and temporal trends of tuberculosis attributable to high sugar-sweetened beverage consumption: insights from the Global Burden of Disease Study 2021

**DOI:** 10.3389/fnut.2025.1638390

**Published:** 2025-10-28

**Authors:** Lijie Qiu, Yixiang Zhang, Kun Yan, Jianxiu Xu, Luxin Fan, Mengmeng Peng, Chengpeng Gao

**Affiliations:** Department of Respiratory Medicine Center, Weifang People’s Hospital, Shandong Second Medical University, Weifang, Shandong, China

**Keywords:** tuberculosis, sugar-sweetened beverage, Global Burden of Disease, disability-adjusted life years (DALYs), Socio-demographic Index (SDI), health inequality, public health

## Abstract

**Background:**

This study aims to assess the current global burden and temporal trends of tuberculosis (TB) attributable to high sugar-sweetened beverage (SSB) consumption, and to analyze its association with the Socio-demographic Index (SDI), in order to provide evidence for TB prevention and control strategies.

**Methods:**

Based on data from the Global Burden of Disease Study 2021 (GBD 2021), we analyzed changes in incidence, prevalence, disability-adjusted life years (DALYs), and mortality of TB attributable to high SSB consumption globally and across regions from 1990 to 2021. The Das Gupta decomposition method was applied to assess the contributions of population growth, aging, and epidemiological changes.

**Results:**

In 2021, global disability-adjusted life years (DALYs) and deaths due to TB attributable to high SSB consumption increased by 52 and 44%, respectively, compared to 1990. However, the age-standardized rates (per 100,000 population) declined. The burden was highest and grew most rapidly in low-middle SDI regions, while high SDI regions experienced the fastest decline in mortality rates. The disease burden peaked in the 50–54 age group and was higher in males than females. Cross-country inequality analysis indicated that the TB burden was more concentrated in lower SDI regions.

**Conclusion:**

The health burden of TB attributable to high SSB consumption presents complex global patterns. Low- and middle-income regions face higher TB risks, highlighting the need for strengthened public health measures, particularly interventions targeting high SSB consumption, to achieve the goal of ending the TB epidemic by 2030.

## Introduction

Tuberculosis (TB) is a chronic infectious disease caused by *Mycobacterium tuberculosis*, primarily affecting the lungs but capable of spreading to other organs through the bloodstream or lymphatic system (1–3). Although the global incidence and mortality rates of TB have declined, the progress remains slow, making it difficult to achieve the World Health Organization’s (WHO) “End TB Strategy” targets. In 2019, an estimated 10 million people developed TB globally, with an incidence rate of 130 per 100,000 population (4). The majority of global TB cases are concentrated in Southeast Asia, Africa, and the Western Pacific. Eight countries—India, Indonesia, China, the Philippines, Pakistan, Nigeria, Bangladesh, and South Africa—accounted for two-thirds of the global total (4).

In terms of treatment and prevention, 7.1 million people received TB treatment in 2019, but 2.9 million cases remained undiagnosed or unreported (5). While the coverage of preventive treatment has improved, disparities in resource allocation persist, particularly in high-burden countries and regions where TB control capacity needs urgent strengthening.

A growing body of evidence has linked the consumption of sugar-sweetened beverages (SSBs) to numerous health issues, including weight gain, obesity, type 2 diabetes, cardiovascular diseases, kidney disease, non-alcoholic fatty liver disease, gout, dental caries, and mental health problems (6–9). The high sugar content in these beverages often leads to excessive caloric intake, insulin resistance, and metabolic dysfunction. Increasing research has shown that SSBs can worsen TB outcomes through multiple mechanisms (10–12). First, large amounts of sucrose and high-fructose corn syrup rapidly elevate blood glucose levels, creating a hyperglycemic microenvironment that directly promotes the proliferation of *Mycobacterium tuberculosis* and significantly increases the risk of diabetes, which in turn raises the probability of TB by 4–8 times. Second, sustained hyperglycemia suppresses T-cell secretion of interferon-γ, weakening macrophage phagocytosis and bactericidal activity, thereby hindering the clearance of TB bacteria. Third, SSBs induce chronic low-grade inflammation and upregulate the expression levels of TNF-α and IL-6, which leads to an expansion of pulmonary lesions and accelerated cavity formation (10). Clinical observations have shown that TB patients with hyperglycemia face a 94% higher risk of sputum smear positivity, experience prolonged time to conversion, and have an increased relapse rate (10).

This study utilizes data from the Global Burden of Disease Study 2021 (GBD 2021) to analyze the temporal trends in the incidence, prevalence, disability-adjusted life years (DALYs), and mortality of TB attributable to high sugar-sweetened beverages consumption (TB attributable to high SSB consumption). By examining data across 21 GBD regions, this study also explores the influence of sex, age, and socio-demographic development on the burden of TB attributable to high SSB consumption. The goal is to serve as a valuable resource for global TB control efforts, providing insights for more effective prevention strategies targeting high-SSB dietary risks and contributing to the global goal of ending the TB epidemic by 2030.

## Methods

### Data sources and disease definition

Data for this study were obtained from the Global Burden of Disease Study 2021 (GBD 2021), which covers 204 countries and territories, 811 subnational regions, 371 diseases and injuries, and 88 risk factors from 1990 to 2021 (13–17). The database provides key health indicators, including incidence, prevalence, mortality, and disability-adjusted life years (DALYs). The Socio-demographic Index (SDI), a composite measure of socioeconomic development, was calculated as the geometric mean of the total fertility rate under age 25 (TFU25), mean years of education in the population aged 15 years or older (EDU15+), and lag-distributed income per capita (LDI), with values ranging from 0 to 1 (18). Tuberculosis (TB), caused by *Mycobacterium tuberculosis* and primarily transmitted through the respiratory route, can affect the lungs as well as other organs, and is coded under ICD-10 A15–A19 (1). The study period spanned from January 1, 1990, to December 31, 2021.

The data extraction process was as follows: We accessed the GBD 2021 online website.^1^ Under the “GBD Estimate” category, we selected “Risk factor.” In the “Measure” category, we chose “Deaths” and “DALYs.” Under the “Metric” category, we selected “Rate” and “Number.” In the “Risk” category, we selected “Diet high in sugar-sweetened beverages.” In the “Cause” category, we selected “Tuberculosis.” For “Location,” we selected “Global,” “5 SDI regions,” “21 GBD regions,” and “204 countries.” Under the “Age” category, we selected “Age-standardized,” “All ages,” and “specific age groups.” In the “Sex” category, we selected “Male,” “Female,” and “Both.” Finally, under the “Year” category, we selected “1990–2021.”

### Disability-adjusted life years

DALYs are a comprehensive measure of disease burden, calculated as the sum of years of life lost due to premature mortality (YLL) and years lived with disability (YLD): DALY = YLL + YLD (16, 19). This metric captures the overall impact of disease on population health and serves as a key reference for public health decision-making.

### Decomposition analysis

This study applied the Das Gupta method to decompose changes in disease burden into contributions from population growth, population aging, and epidemiological changes (18, 20, 21). This approach clarifies the role of each factor in the overall change and provides quantitative support for public health interventions.

### Cross-country inequality analysis

Cross-national inequality analysis evaluates health and socioeconomic disparities between countries (13, 14, 22). Slope index of inequality (SII) is used to assess absolute inequality, while the concentration index (CI) reflects how the health burden is distributed across countries with varying SDI levels. A positive value indicates a higher burden concentrated in high SDI countries, whereas a negative value indicates the opposite. This analysis helps identify health disparities and supports global efforts toward equitable development.

### Forecasting analysis

This study used the Nordpred model to forecast the future burden of TB (20). The model incorporates historical trends and current demographic structures to estimate future incidence levels, providing a basis for public health planning and resource allocation.

### Statistical analysis

The estimated annual percentage change (EAPC) is a statistical indicator used in epidemiological and public health research to measure the average annual change in a health indicator (such as DALYs or mortality rate) over a specific period of time (18, 22). The calculation is usually based on a log-linear regression model, with year as the independent variable and the natural logarithm of the indicator as the dependent variable. The fitted equation is log(*Y*) = *α* + *β*·*t* + *ε*, where *Y* represents the age-standardized rate, *t* represents the time variable, *α* is the intercept, *β* is the coefficient of the time variable, and *ε* is the error term. The EAPC is derived from the formula 100 × (exp(*β*) − 1) (23). In addition, this study also used percentage change to reflect the change in the number of DALYs and death cases in 2021 compared with 1990. Percentage change = (2021 cases − 1990 cases)/1990 cases.

Moreover, the age-standardized mortality rate (ASMR), age-standardized DALY rate (ASDR), and their corresponding EAPC and percentage change were used to compare the disease burden and time trends across different genders, ages, and geographical regions. The Nordpred R package was applied for predictive analysis. Data cleaning, computation, and graph plotting were conducted using R software (version 4.2.1) in this study.

## Results

### Global and regional burden of TB attributable to high SSB consumption

**Figure 1:**
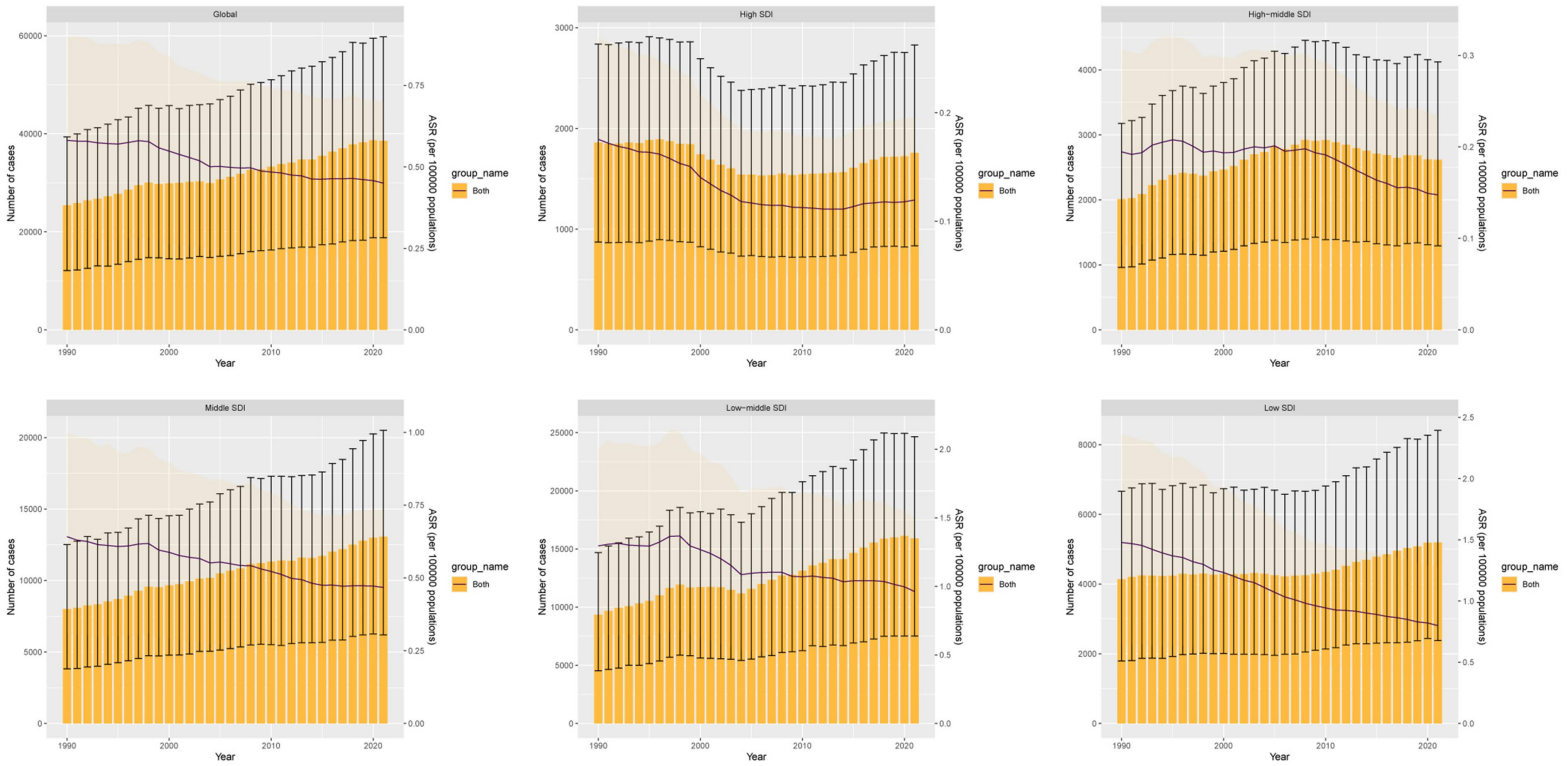
DALYs cases and ASDR of TB attributable to high SSB consumption from 1990 to 2021.

In 2021, the number of DALYs cases of TB attributable to high SSB consumption was 38576.23, a 52% increase from 25390.76 in 1990. However, the ASDR decreased from 0.58 per 100,000 in 1990 to 0.45 per 100,000 in 2021, with an EAPC of −0.94 (95% CI, −1.02 to −0.86). In 2021, there were 1067.92 death cases, a 44% increase from 739.4 in 1990. The ASMR decreased from 0.01806 per 100,000 in 1990 to 0.01246 per 100,000 in 2021, with an EAPC of −1.33 (95% CI, −1.41 to −1.24) (Figure 1, Supplementary Figure 1).

Among the five SDI regions, the low-middle SDI region had the highest DALYs and death cases, at 15920.15 and 445.92 respectively, with percentage changes of 70 and 63%. This region also recorded the highest ASDR and ASMR at 0.96 and 0.02963 per 100,000 respectively, with EAPCs of −1.08 (95% CI: −1.21 to −0.94) and −1.3 (95% CI: −1.43 to −1.16). Over the past 32 years, the low SDI region experienced the fastest decline in ASDR, with an EAPC of −2.1 (95% CI: −2.19 to −2.02), while the high SDI region saw the steepest decline in ASMR, with an EAPC of −2.65 (95% CI: −2.92 to −2.37). From 1990 to 2021, Western Sub-Saharan Africa experienced the fastest increase in DALYs and death cases among the 21 GBD regions, with percentage changes of 261 and 237%, and EAPCs of 1.61 (95% CI, 1.18 to 2.05) and 1.55 (95% CI, 1.12 to 1.98), respectively (Tables 1, 2).

**Table 1:** Disability-adjusted life years (DALYs) and age-standardized DALY rate (ASDR) of TB attributable to high SSB consumption in 1990 and 2021, and the PC and EAPC from 1990 to 2021.

Location	1990_DALYs cases (95% UI)	2021_DALYs cases (95% UI)	Percentage change	1990_ASDR_per 100,000 (95% UI)	2021_ASDR_per 100,000 (95% UI)	EAPC (95% CI)
Andean Latin America	360.94 (160.2–592.82)	288.87 (136.59–475.73)	−0.2	1.49 (0.66–2.4)	0.46 (0.22–0.75)	−3.83 (−4.23 to −3.42)
Australasia	13.9 (6.67–22.47)	13.36 (6.1–20.97)	−0.04	0.06 (0.03–0.1)	0.03 (0.01–0.04)	−2.47 (−2.89 to −2.04)
Caribbean	64.33 (28.58–114.65)	97.22 (42.59–172.01)	0.51	0.22 (0.1–0.4)	0.19 (0.08–0.34)	0.22 (−0.15 to 0.6)
Central Asia	143.07 (65.13–239.17)	233.18 (108.21–388.41)	0.63	0.26 (0.12–0.43)	0.23 (0.11–0.39)	−1.19 (−1.91 to −0.47)
Central Europe	515.09 (234.68–812.35)	401.13 (197.99–642.64)	−0.22	0.35 (0.16–0.55)	0.23 (0.11–0.38)	−1.06 (−1.25 to −0.86)
Central Latin America	1557.73 (710.86–2494.16)	1044.97 (471.6–1691.55)	−0.33	1.47 (0.68–2.35)	0.39 (0.18–0.63)	−4.32 (−4.75 to −3.89)
Central Sub-Saharan Africa	1719.76 (720.58–3215.85)	1846.54 (741.4–3384.05)	0.07	5.71 (2.39–10.36)	2.29 (0.94–4.11)	−2.94 (−3.21 to −2.68)
East Asia	1234.13 (580.3–1947.49)	1682.55 (812.23–2724.35)	0.36	0.13 (0.06–0.2)	0.08 (0.04–0.13)	−1.28 (−1.43 to −1.12)
Eastern Europe	316.78 (146.37–509.78)	369.59 (172.65–607.21)	0.17	0.12 (0.05–0.19)	0.13 (0.06–0.22)	0.61 (−0.55 to 1.78)
Eastern Sub-Saharan Africa	891.27 (404.44–1453.41)	1613.06 (728.52–2692.52)	0.81	1 (0.47–1.59)	0.75 (0.35–1.24)	−0.89 (−0.98 to −0.79)
Global	25390.76 (12097.18–39381.26)	38576.23 (18808.79–59787.41)	0.52	0.58 (0.28–0.9)	0.45 (0.22–0.7)	−0.94 (−1.02 to −0.86)
High-income Asia Pacific	659.97 (308.68–1012.64)	366.78 (182.05–573.7)	−0.44	0.33 (0.15–0.5)	0.08 (0.04–0.13)	−4.93 (−5.24 to −4.63)
High-income North America	345.25 (149.73–561.02)	250.33 (118.72–388.52)	−0.27	0.1 (0.04–0.17)	0.05 (0.02–0.08)	−3.15 (−3.54 to −2.75)
High-middle SDI	2013.04 (962.02–3178.33)	2618.75 (1293.96–4123.91)	0.3	0.19 (0.09–0.31)	0.15 (0.07–0.23)	−0.93 (−1.15 to −0.72)
High SDI	1860.47 (872.31–2839.02)	1759.1 (834.13–2828.7)	−0.05	0.18 (0.08–0.27)	0.12 (0.05–0.2)	−1.51 (−1.81 to −1.21)
Low-middle SDI	9354.59 (4539.52–14683.84)	15920.15 (7520.06–24644.72)	0.7	1.3 (0.63–2.01)	0.96 (0.46–1.49)	−1.08 (−1.21 to −0.94)
Low SDI	4138.01 (1794.22–6666.37)	5194.33 (2380.93–8412.09)	0.26	1.48 (0.65–2.36)	0.8 (0.38–1.28)	−2.1 (−2.19 to −2.02)
Middle SDI	8007.43 (3816.53–12521.01)	13063.98 (6188.97–20503.77)	0.63	0.64 (0.31–1)	0.47 (0.22–0.73)	−1.12 (−1.18 to −1.05)
North Africa and Middle East	866.43 (389.42–1479.41)	1603.67 (723.34–2627.34)	0.85	0.43 (0.2–0.74)	0.27 (0.12–0.44)	−1.57 (−1.73 to −1.4)
Oceania	52.39 (22.83–89.27)	81.2 (37.29–127.55)	0.55	1.3 (0.58–2.21)	0.8 (0.36–1.28)	−1.85 (−1.95 to −1.75)
South Asia	13126.26 (6340.8–20619.99)	21491.61 (10363–33935.63)	0.64	1.88 (0.91–2.94)	1.27 (0.61–1.98)	−1.54 (−1.71 to −1.37)
Southeast Asia	1337.35 (621.2–2102.78)	2901.13 (1396.88–4567.25)	1.17	0.46 (0.22–0.71)	0.41 (0.2–0.64)	−0.33 (−0.39 to −0.28)
Southern Latin America	245.34 (112.72–397.88)	271.24 (127.36–431.79)	0.11	0.52 (0.24–0.85)	0.34 (0.16–0.54)	−1.41 (−1.61 to −1.2)
Southern Sub-Saharan Africa	656.8 (277.55–1098.5)	1663.72 (731.87–2705.95)	1.53	1.82 (0.8–2.99)	2.29 (1.03–3.71)	1.46 (0.52–2.39)
Tropical Latin America	472.95 (220.2–760.92)	913.37 (388.74–1460.03)	0.93	0.41 (0.19–0.65)	0.35 (0.15–0.55)	−0.55 (−0.73 to −0.38)
Western Europe	463.96 (227.46–719.86)	189.45 (91.46–292.14)	−0.59	0.09 (0.04–0.14)	0.02 (0.01–0.04)	−4.41 (−4.54 to −4.27)
Western Sub-Saharan Africa	347.08 (170.87–553.96)	1253.25 (555.39–1987.53)	2.61	0.35 (0.17–0.56)	0.52 (0.23–0.83)	1.61 (1.18–2.05)

**Table 2:** Deaths and age-standardized mortality rate (ASMR) of TB attributable to high SSB consumption in 1990 and 2021, and the PC and EAPC from 1990 to 2021.

Location	1990_Death cases (95% UI)	2021_Death cases (95% UI)	Percentage change	1990_ASMR_per 100,000 (95% UI)	2021_ASMR_per 100,000 (95% UI)	EAPC (95% CI)
Andean Latin America	10.5 (4.79–16.73)	8.39 (4.04–13.6)	−0.2	0.04783 (0.02249–0.07497)	0.01377 (0.00664–0.02228)	−3.99 (−4.38 to −3.59)
Australasia	0.59 (0.27–0.94)	0.58 (0.26–0.94)	−0.02	0.00251 (0.00117–0.004)	0.00103 (0.00047–0.00164)	−2.87 (−3.29 to −2.46)
Caribbean	1.68 (0.73–3.21)	2.41 (1.06–4.42)	0.43	0.00616 (0.00271–0.01166)	0.00462 (0.00203–0.00845)	−0.22 (−0.61 to 0.18)
Central Asia	3.43 (1.59–5.63)	5.35 (2.5–8.82)	0.56	0.00662 (0.00312–0.01069)	0.00558 (0.00263–0.00912)	−1.32 (−1.98 to −0.65)
Central Europe	16.56 (7.62–25.63)	11.92 (6.13–18.84)	−0.28	0.0113 (0.0052–0.01748)	0.00631 (0.00316–0.01)	−1.64 (−1.83 to −1.45)
Central Latin America	41.56 (19.31–66.05)	25.38 (11.94–40.27)	−0.39	0.04444 (0.021–0.07036)	0.00974 (0.0046–0.0154)	−5 (−5.43 to −4.56)
Central Sub-Saharan Africa	43.99 (18.52–80.96)	44.87 (18.4–81.09)	0.02	0.17443 (0.07131–0.31244)	0.06786 (0.02796–0.12112)	−3.07 (−3.3 to −2.83)
East Asia	38.45 (19.11–60.95)	44.82 (21.66–74.46)	0.17	0.00473 (0.00239–0.00742)	0.00214 (0.00103–0.00353)	−2.53 (−2.69 to −2.36)
Eastern Europe	8.23 (3.76–12.99)	9.28 (4.4–15.02)	0.13	0.00298 (0.00137–0.00469)	0.00311 (0.00148–0.00514)	0.41 (−0.68 to 1.5)
Eastern Sub-Saharan Africa	26.56 (12.48–42.33)	46.82 (21.37–76.71)	0.76	0.03691 (0.01752–0.05784)	0.02743 (0.01268–0.04334)	−0.92 (−1.01 to −0.83)
Global	739.4 (355.33–1133.15)	1067.92 (512.38–1643.54)	0.44	0.01806 (0.00875–0.02742)	0.01246 (0.00597–0.01917)	−1.33 (−1.41 to −1.24)
High-income Asia Pacific	26.77 (12.56–40.63)	22.63 (11.26–36.85)	−0.15	0.01386 (0.00646–0.02101)	0.00382 (0.0019–0.006)	−4.65 (−4.92 to −4.38)
High-income North America	12.38 (5.54–19.91)	7.71 (3.77–11.9)	−0.38	0.00357 (0.00158–0.0057)	0.00136 (0.00066–0.0021)	−4.03 (−4.47 to −3.59)
High-middle SDI	61.27 (29.3–94.18)	71.5 (35.04–111.35)	0.17	0.00626 (0.00302–0.00953)	0.00384 (0.00187–0.00594)	−1.64 (−1.81 to −1.46)
High SDI	69.22 (32.9–103.49)	58.97 (28.68–91.23)	−0.15	0.00633 (0.00301–0.00945)	0.00319 (0.00154–0.00502)	−2.65 (−2.92 to −2.37)
Low-middle SDI	272.86 (132.24–424.91)	445.92 (206.71–691.06)	0.63	0.04229 (0.02059–0.06569)	0.02963 (0.01371–0.04634)	−1.3 (−1.43 to −1.16)
Low SDI	114.11 (51.29–182.97)	140.6 (66.79–223.12)	0.23	0.04705 (0.02162–0.07473)	0.02585 (0.01235–0.04102)	−2.05 (−2.13 to −1.97)
Middle SDI	221.42 (107.11–339.8)	350.38 (166.29–550.36)	0.58	0.02034 (0.00991–0.03072)	0.01289 (0.00612–0.02023)	−1.55 (−1.62 to −1.48)
North Africa and Middle East	25.34 (11.23–43.53)	38.26 (17.57–62.2)	0.51	0.01508 (0.00663–0.02663)	0.00739 (0.00343–0.01211)	−2.33 (−2.47 to −2.18)
Oceania	1.3 (0.58–2.2)	1.97 (0.88–3.1)	0.52	0.03932 (0.01773–0.06672)	0.02357 (0.01056–0.03726)	−1.9 (−2.01 to −1.79)
South Asia	374.65 (180.96–585.47)	588.46 (278.6–920.66)	0.57	0.06039 (0.02935–0.09367)	0.03801 (0.01801–0.05942)	−1.81 (−1.99 to −1.63)
Southeast Asia	41.27 (19.6–62.97)	88.55 (42.26–135.33)	1.15	0.01688 (0.00814–0.02597)	0.01403 (0.00671–0.02147)	−0.51 (−0.6 to −0.42)
Southern Latin America	7.78 (3.55–12.32)	8.39 (4.03–13.14)	0.08	0.01688 (0.00775–0.02663)	0.01002 (0.00479–0.01572)	−1.74 (−1.93 to −1.54)
Southern Sub-Saharan Africa	15.69 (6.81–25.77)	42.2 (19.33–66.63)	1.69	0.04963 (0.02202–0.08074)	0.06565 (0.03088–0.10368)	1.57 (0.7–2.44)
Tropical Latin America	11.75 (5.39–18.67)	23.23 (10.44–36.12)	0.98	0.01124 (0.00521–0.01772)	0.00889 (0.00399–0.01381)	−0.83 (−0.99 to −0.66)
Western Europe	19.84 (9.9–30.55)	9.25 (4.51–14.46)	−0.53	0.00344 (0.0017–0.0053)	0.00091 (0.00044–0.0014)	−4.67 (−4.83 to −4.5)
Western Sub-Saharan Africa	11.1 (5.49–17.63)	37.45 (16.62–58.87)	2.37	0.01323 (0.00654–0.02134)	0.01949 (0.0089–0.03037)	1.55 (1.12–1.98)

### National burden of TB attributable to high SSB consumption

**Figure 2:**
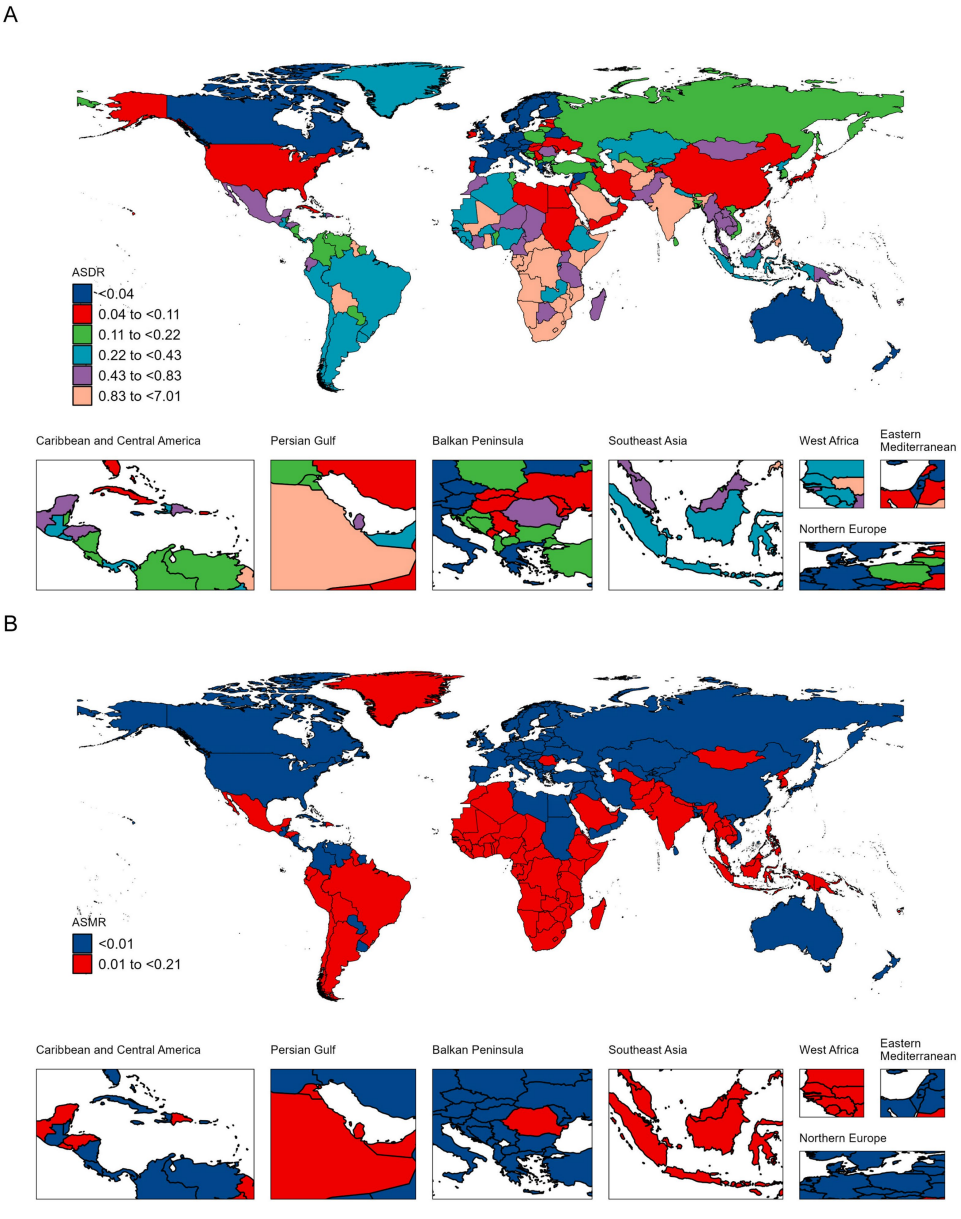
ASDR **(A)** and ASMR **(B)** of TB attributable to high SSB consumption per 100,000 population in 2021, by country.

Over the past 32 years, the top five countries with the fastest-growing DALYs cases were Ghana, Equatorial Guinea, Djibouti, Turkmenistan, and Lesotho, with percentage changes of 960, 608, 576, 493, and 454%, respectively. The top five countries with the fastest-growing death cases were Ghana, Djibouti, Turkmenistan, Equatorial Guinea, and Kuwait, with increases of 909, 550, 447, 431, and 425%, respectively. The top five countries with the fastest decline in ASDR were Cyprus, Maldives, Japan, Hungary, and Ecuador, with EAPCs of −6.72 (95% CI: −7 to −6.44), −6.65 (95% CI: −7.14 to −6.15), −6.1 (95% CI: −6.41 to −5.79), −6.09 (95% CI: −6.29 to −5.9), and −5.72 (95% CI: −6.07 to −5.36), respectively. The top five countries with the fastest decline in ASMR were Cyprus, Bermuda, Hungary, Taiwan, and Maldives, with EAPCs of −7.13 (95% CI: −7.54 to −6.71), −6.84 (95% CI: −7.51 to −6.17), −6.68 (95% CI: −6.89 to −6.48), −6.59 (95% CI: −7.02 to −6.16), and −6.47 (95% CI: −6.88 to −6.06), respectively (Figures 2A,B, Supplementary Tables 1, 2).

### Age and sex differences in the burden of TB attributable to high SSB consumption

**Figure 3:**
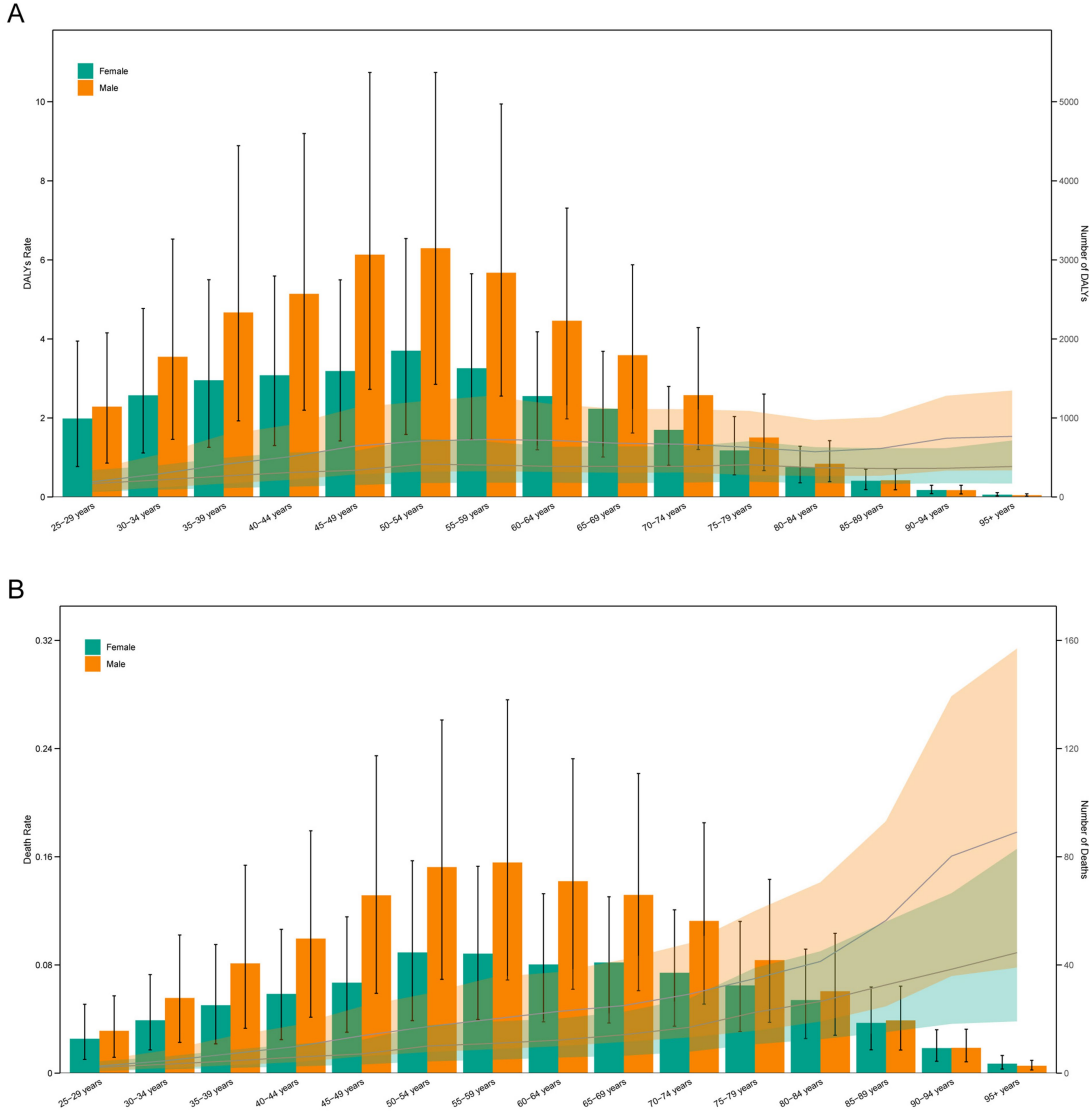
Age-specific numbers and rates of DALYs **(A)** and deaths **(B)** of TB attributable to high SSB consumption by age and sex in 2021.

In 2021, DALYs cases of TB attributable to high SSB consumption increased progressively with age, peaking in the 50–54 age group. Among individuals under 90 years, men had significantly higher DALYs cases than women. Additionally, male death cases peaked in the 55–59 age group, while female death cases peaked in the 50–54 age group. The highest DALYs and death rates for men occurred in the 95+ age group, while for women, the highest DALYs rates was in the 50–54 age group and the highest death rates was in the 95+ age group (Figures 3A,B).

### Relationship between the burden of TB attributable to high SSB consumption and SDI

**Figure 4:**
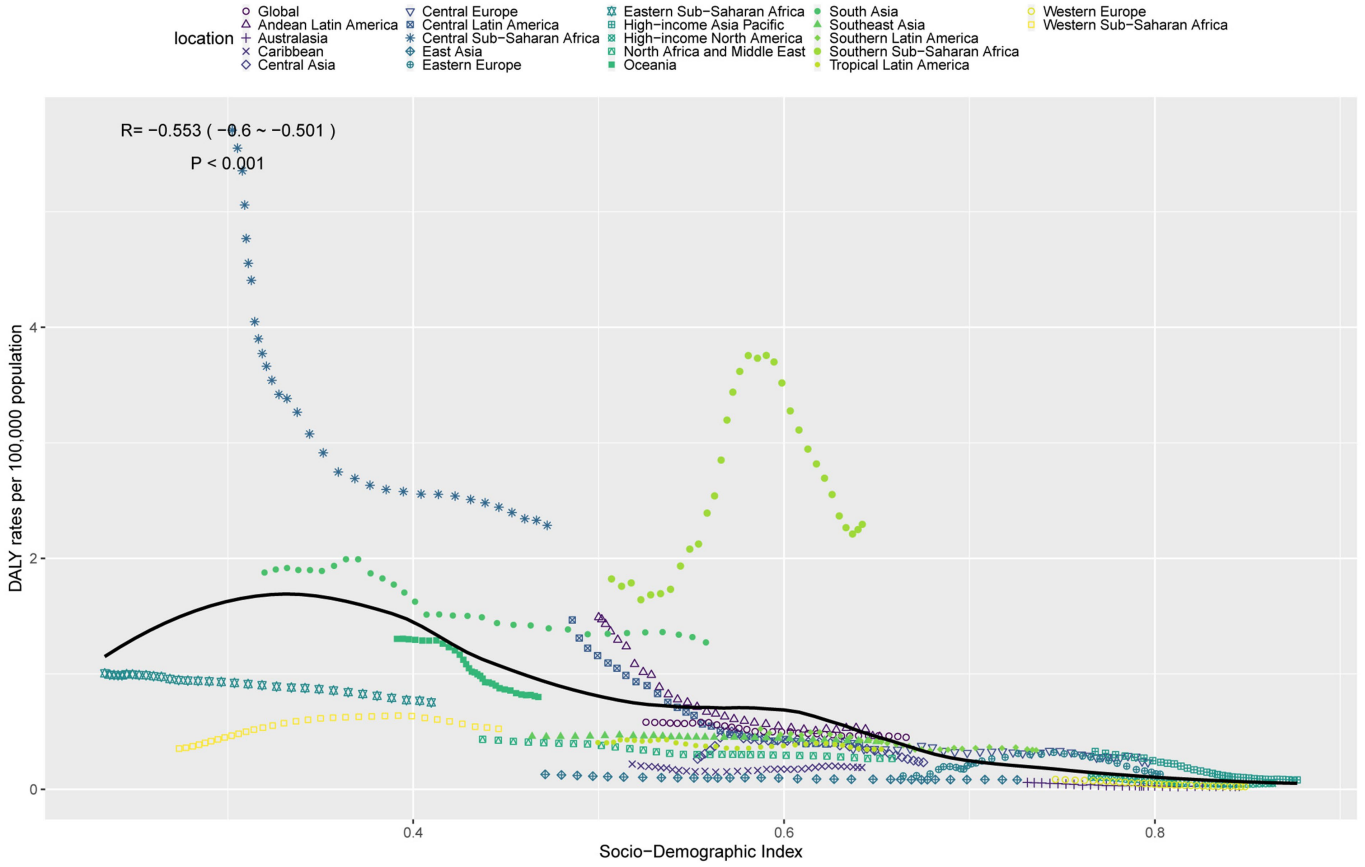
ASDR of TB attributable to high SSB consumption in 21 GBD regions by SDI, 1990–2021.

In 2021, there was a significant negative correlation between ASDR and ASMR for TB attributable to high SSB consumption and the SDI (Figure 4, Supplementary Figure 2). With economic development, the overall disease burden showed a decreasing trend. Specifically, when the SDI exceeded 0.4, ASDR and ASMR declined gradually with increasing SDI. Notably, the disease burden in Central Sub-Saharan Africa, South Asia, and Southern Sub-Saharan Africa was significantly higher than expected, while that in Eastern Sub-Saharan Africa, Western Sub-Saharan Africa, North Africa and Middle East, East Asia, and Caribbean was significantly lower than expected.

### Decomposition analysis of TB burden attributable to high SSB consumption

**Figure 5:**
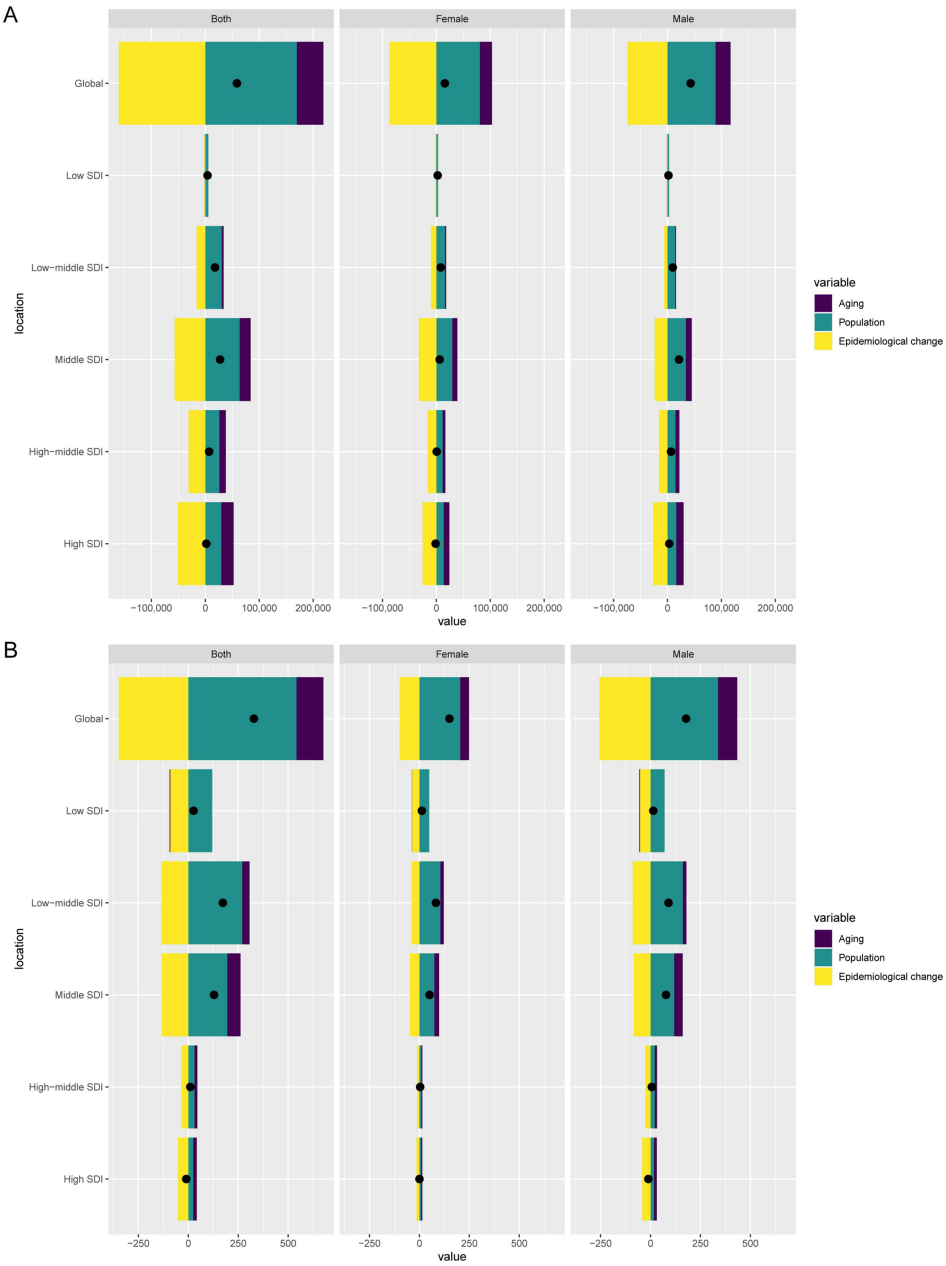
Decomposition analysis of changes in DALYs **(A)** and deaths **(B)** of TB attributable to high SSB consumption between 1990 and 2021 across SDI regions.

Over the past 32 years, global DALYs cases increased by 13185.47, of which aging contributed 2534.19 (19.22%), population growth contributed 18961.28 (143.8%), and epidemiological changes contributed −8,310 (−63.02%). In high SDI regions, DALYs cases decreased by 101.37, with aging contributing 207.35 (−204.55%), population growth contributing 676.75 (−667.62%), and epidemiological changes contributing −985.46 (972.17%) (Figure 5A).

Globally, death cases increased by 328.52, with aging contributing 134.79 (41.03%), population growth contributing 541.87 (164.94%), and epidemiological changes contributing −348.14 (−105.97%). In high SDI regions, death cases decreased by 10.24, with aging contributing 17.84 (−174.17%), population growth contributing 24.36 (−237.84%), and epidemiological changes contributing −52.44 (512%) (Figure 5B).

### Cross-country inequality in TB burden attributable to high SSB consumption

**Figure 6:**
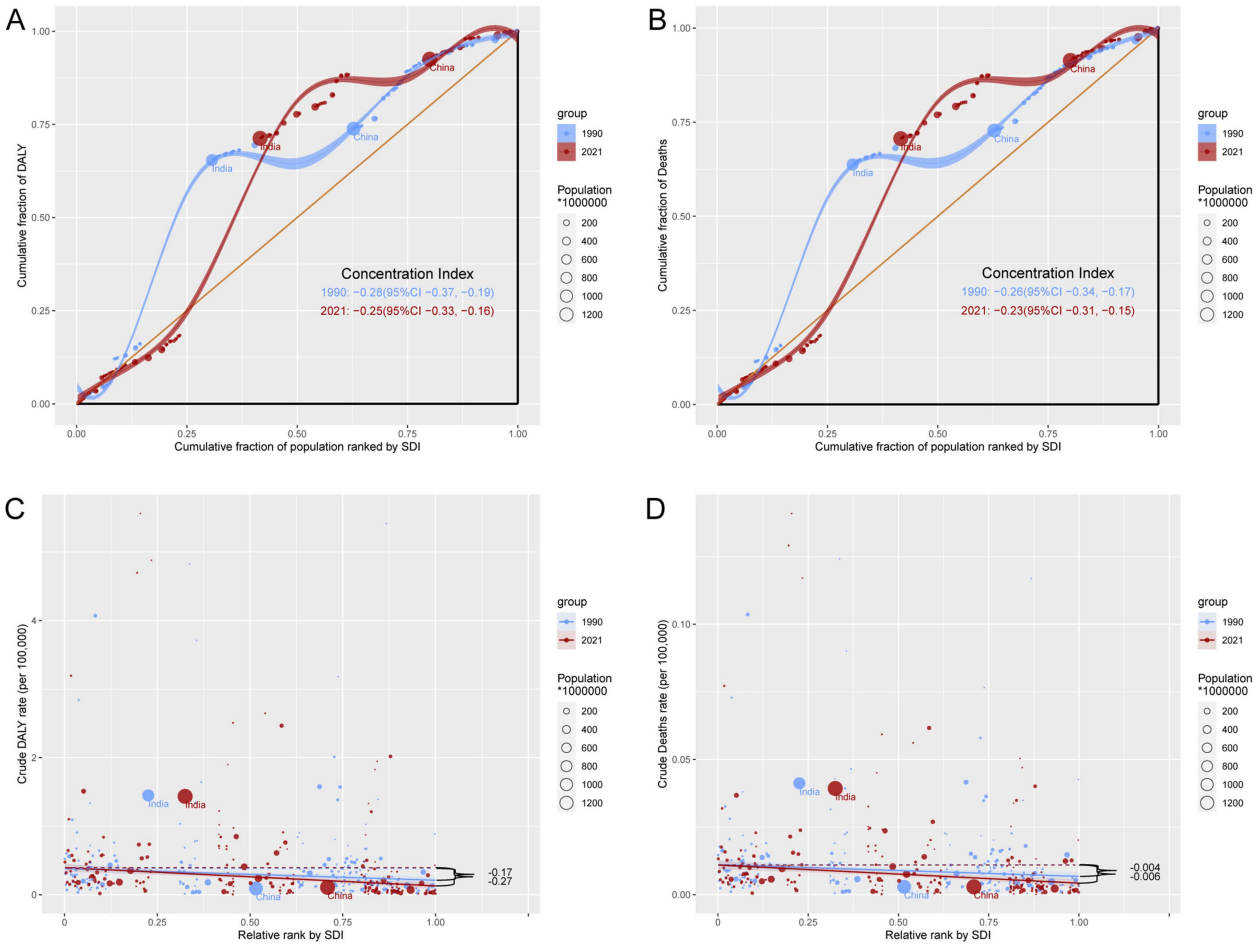
Health inequality analysis of DALYs and mortality in TB attributable to high SSB consumption in 1990 and 2021 across the world. **(A)** Concentration curves for DALYs. **(B)** Concentration curves for mortality. **(C)** Health inequality regression curves for DALYs. **(D)** Health inequality regression curves for mortality.

From 1990 to 2021, the SII for DALYs rates increased from −0.28 to −0.25, and for death rates from −0.26 to −0.23, indicating a narrowing gap in DALYs and death rates between the highest and lowest SDI regions. However, the CI for DALYs rates decreased from −0.17 in 1990 to −0.27 in 2021, and for death rates from −0.004 to −0.006. Thus, the burden of TB attributable to high SSB consumption has become increasingly concentrated in lower SDI regions, with a growing trend of relative inequality (Figures 6A–D).

### Forecast analysis of TB burden attributable to high SSB consumption

**Figure 7:**
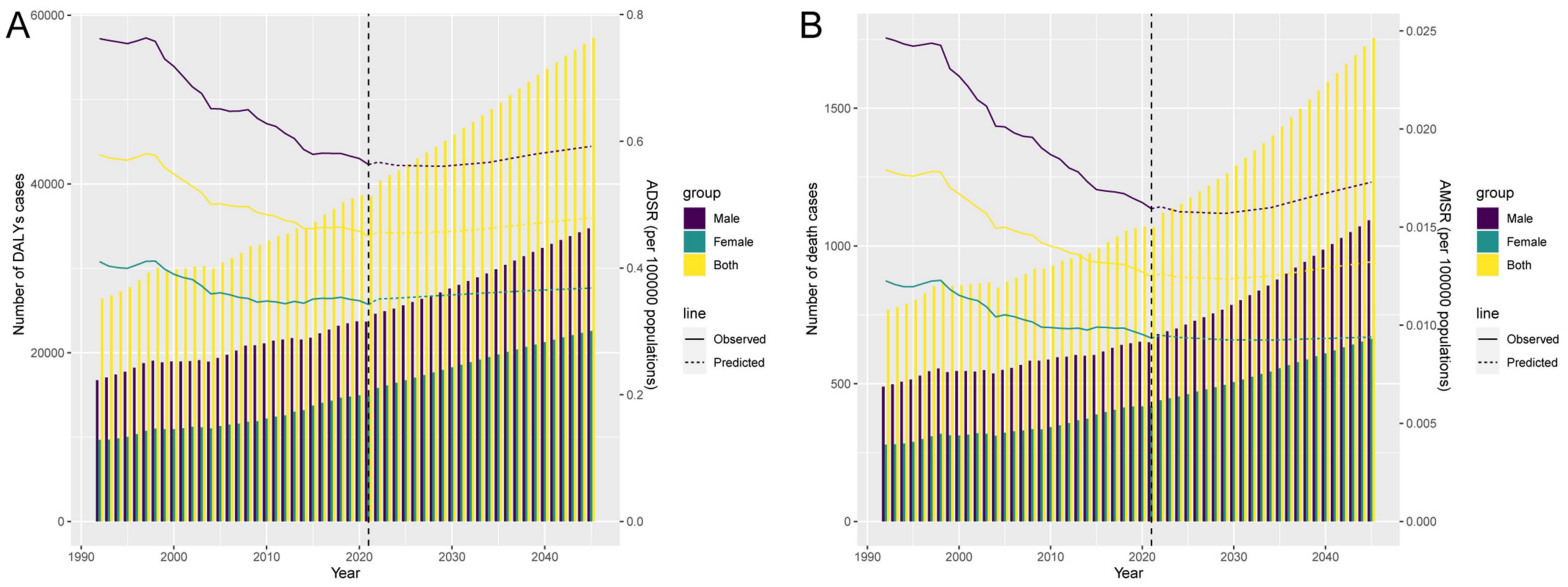
Projections of the temporal trends of the number of DALYs cases, mortality cases, ASDR, and ASMR of TB attributable to high SSB consumption globally up to 2045. **(A)** The number and ASDR of TB attributable to high SSB consumption by year and gender. **(B)** The number and ASMR of TB attributable to high SSB consumption by year and gender.

Forecast analysis suggests that from 2022 to 2045, the numbers of DALYs and death cases will continue to rise annually, with male cases consistently higher than female cases. Notably, while ASDR and ASMR showed significant declines over the past 30 years, they are projected to remain relatively stable or slightly increase in the next two decades, with future ASDR and ASMR expected to be significantly higher in males than in females (Figures 7A,B).

## Discussion

The study found that SSBs contain large amounts of absorbable carbohydrates, which can cause rapid increases in blood glucose and insulin concentrations (24, 25). Long-term and excessive consumption raises dietary glycemic load, leading to impaired glucose tolerance and insulin resistance (26, 27). It also promotes hepatic fat synthesis, resulting in hypertriglyceridemia and other metabolic abnormalities. SSB intake is closely associated with inflammatory responses, as fructose can activate inflammatory pathways, trigger chronic inflammation, and increase the risk of insulin resistance and type 2 diabetes (28, 29). There is a metabolic and immune interaction between type 2 diabetes and tuberculosis. A hyperglycemic environment is conducive to the growth of *Mycobacterium tuberculosis* and can impair host defense mechanisms. SSB intake may indirectly weaken anti-tuberculosis immunity by increasing the risk of type 2 diabetes (28).

From 1990 to 2021, the impact of SSB consumption on the TB burden showed complex and multidimensional changes. Although the absolute numbers of DALYs and deaths due to TB attributable to high SSB consumption have continued to rise globally, the rates (per 100,000 population) have declined. This trend indicates that while population growth has driven the absolute increase in cases, the relative impact has weakened with improvements in overall health and the strengthening of public health interventions. Such changes may partly be attributed to ongoing global TB control efforts such as screening, standardized treatment, and vaccination programs (30). However, metabolic risks induced by SSB consumption continue to exacerbate the disease burden.

Marked disparities were observed across regions with different SDI levels. Low-middle SDI regions showed the highest DALYs and deaths from TB, with relatively rapid growth, reflecting the combined effects of insufficient medical resources, limited health education, and imbalanced nutrition. Although low SDI regions experienced the fastest decline in DALYs rates, their heavy burden still makes them a priority for global TB control. This progress can be partly credited to recent international aid and investment in public health programs. In contrast, high SDI regions achieved the most substantial reductions in mortality rates, benefiting from well-developed healthcare systems and higher health awareness. From the GBD regional perspective, Western Sub-Saharan Africa experienced the fastest growth in TB DALYs and deaths, closely linked to social unrest, poverty, disrupted nutrition, and fragile health systems, highlighting severe global health inequities (31).

At the national level, disparities were equally striking. Over the past 32 years, Ghana, Equatorial Guinea, and Djibouti recorded the fastest increases in DALYs, while Cyprus, Maldives, and Japan saw the steepest declines in ASDR and ASMR, suggesting highly uneven progress in TB control across countries. Age and sex differences further revealed the profound impact of SSB consumption on TB burden. In 2021, TB DALYs peaked among people aged 50–54 years, with men consistently carrying a higher burden than women in older age groups. This is closely associated with men’s generally less healthy lifestyles (such as higher smoking and alcohol consumption), occupational exposures, and the cumulative effects of metabolic disorders (32). TB deaths peaked among women aged 50–54 years and men aged 55–59 years, suggesting that interactions between age and sex play a critical role in disease progression. SDI levels were significantly negatively correlated with TB burden, but actual disease burden in Central Sub-Saharan Africa, South Asia, and Southern Sub-Saharan were much higher than predicted, underscoring the key role of economic, social, and health system vulnerabilities.

Overall, during the past 32 years, the global burden of TB attributable to high SSB consumption has been characterized by increasing absolute numbers but declining relative risk. Population growth has been the main driver of rising DALYs and deaths, while favorable epidemiological changes have partially offset this trend. In high SDI regions, control measures have yielded positive outcomes, with DALYs and deaths showing a decline. However, stark cross-country inequalities persist, with the burden concentrated in low SDI regions, where the gap continues to widen. Predictive analysis indicated that from 2022 to 2045, DALYs and deaths will continue to increase, with male cases consistently exceeding female cases, while ASDR and ASMR are expected to remain stable or rise slightly. This suggests that although global control measures have improved the situation to some extent, regional disparities, sex and age differences, and modifiable risk factors such as diet and lifestyle remain major determinants of the future TB burden. Notably, in 2014, Mexico implemented a 10% excise tax on SSBs, which led to a 7.6% reduction in sales within 2 years. Modeling studies suggest that this policy could prevent about 200,000 cases of obesity and save nearly 1 billion USD in healthcare costs over 10 years (33). Similarly, raising SSB taxes in other countries could reduce consumption, lower TB risk, and substantially cut healthcare expenditures.

There may be some potential confounding factors that have not been identified or adequately considered in this study when assessing the relationship between SSB intake and the burden of TB. Individual lifestyle factors (such as smoking, alcohol consumption, and physical inactivity), socioeconomic status, access to healthcare, and genetic predispositions may all influence TB onset and progression and could correlate with SSB consumption (34–36). Future research should involve more individual-level cohort studies with long-term follow-up and detailed personal data collection to more accurately assess the causal relationship between SSB intake and TB, while better identifying and controlling for potential confounders.

This study also has several limitations. First, it relies on data from the GBD 2021 database. Although the GBD is a key data source for global health research, the integrity and accuracy of its data may be affected by the quality of reporting from different countries and regions. Second, the measurement of SSB intake may lack precision. Consumers may underreport or overreport their consumption, leading to data bias.

## Conclusion

This study, based on GBD 2021, reveals the significant impact of SSB consumption on the global TB burden. While the absolute numbers of DALYs and death cases for TB attributable to high SSB consumption increased, ASDR and ASMR showed a declining trend. The disease burden was heaviest and grew most rapidly in low-middle SDI regions, whereas high SDI regions experienced the fastest declines in mortality rates, reflecting ongoing global health inequalities. In addition, substantial differences in the burden of TB attributable to high SSB consumption were observed by age and sex, with the highest burden seen in the 50–54 age group. Cross-country inequality analysis showed that the burden of TB attributable to high SSB consumption is increasingly concentrated in lower SDI regions, with worsening relative inequality. Forecasting analyses indicate that the TB burden may continue to rise over the next two decades, posing a serious challenge for global public health. This underscores the urgent need to strengthen preventive strategies, particularly those targeting SSB consumption. Specifically, fiscal policies such as taxation could be used to raise the price of SSBs and thereby curb consumption. At the same time, large-scale public health campaigns are needed to raise awareness of the risks associated with SSB intake and promote healthier dietary practices. Furthermore, the vigorous implementation of healthy eating policies in schools and workplaces can also substantially reduce SSB consumption. Collectively, these measures may help mitigate the rising TB burden and advance global health equity.

## Data Availability

The original contributions presented in the study are included in the article/Supplementary material, further inquiries can be directed to the corresponding author.

## Footnotes

1
http://ghdx.healthdata.org/gbd-results-tool
