# Correction: Global burden and temporal trends of tuberculosis attributable to high sugar-sweetened beverage consumption: insights from the Global Burden of Disease Study 2021

**DOI:** 10.3389/fnut.2025.1739086

**Published:** 2025-12-05

**Authors:** Lijie Qiu, Yixiang Zhang, Kun Yan, Jianxiu Xu, Luxin Fan, Mengmeng Peng, Chengpeng Gao

**Affiliations:** Department of Respiratory Medicine Center, Weifang People's Hospital, Shandong Second Medical University, Weifang, Shandong, China

**Keywords:** tuberculosis, sugar-sweetened beverage, Global Burden of Disease, disability-adjusted life years (DALYs), Socio-demographic Index (SDI), health inequality, public health

There was a mistake in Figure 4 as published. Figure 4 did not include the lower half of the image. The corrected Figure 4 appears below.

**Figure 4:**
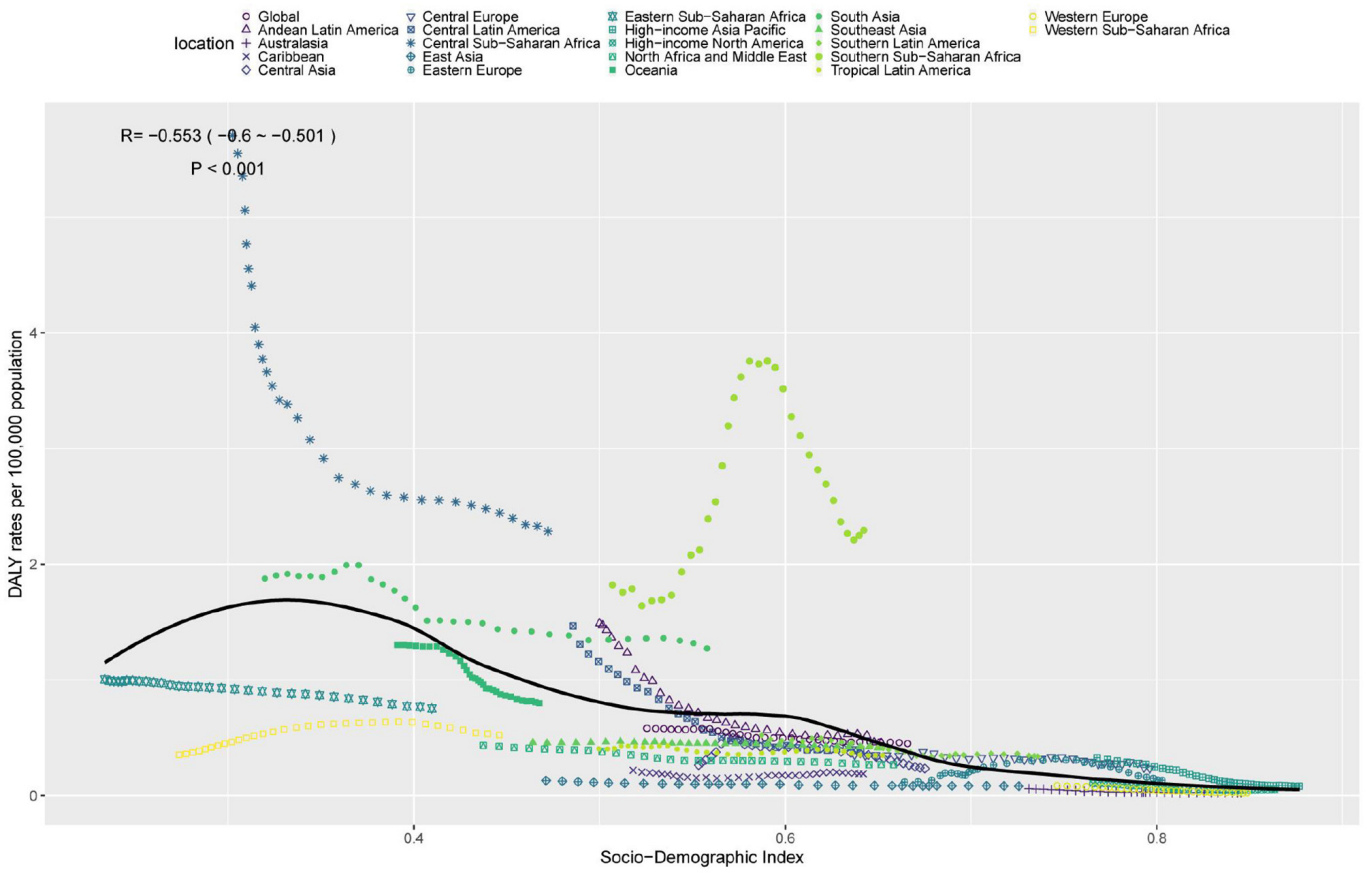
ASDR of TB attributable to high SSB consumption in 21 GBD regions by SDI, 1990–2021.

The original version of this article has been updated.

