# Supplementary material for: Global burden and temporal trends of tuberculosis attributable to high sugar-sweetened beverage consumption: insights from the Global Burden of Disease Study 2021
Source: Front Nutr. 2025 Oct 28;12:1638390. doi: 10.3389/fnut.2025.1638390 (PMC12602238; doi:10.3389/fnut.2025.1638390)
Supplement: Supplementary file 1 [file Table_1.pdf]

Location	1990_DALYs cases (95% UI)	2021_DALYs cases (95% UI)	Percentage change	1990_ASDR_per 100000(95% UI)	2021_ASDR_per 100 000(95% UI)	EAPC (95% CI)
Afghanistan	244.54 (66.99-483.21)	1037.55 (344.32-2004.16)	3.24	2.46 (0.67-4.86)	3.32 (1.1-6.42)	1.77 (0.89-2.67)
Albania	39.38 (17.12-62.62)	36.04 (14.15-62.05)	-0.08	1.19 (0.52-1.89)	1.35 (0.53-2.33)	-3.65 (-4.12--3.18)
Algeria	152.66 (70.19-237.14)	123.02 (49.11-210.34)	-0.19	0.6 (0.28-0.94)	0.28 (0.11-0.48)	-4.8 (-5.3--4.3)
American Samoa	0.13 (0.06-0.23)	0.1 (0.03-0.2)	-0.23	0.28 (0.12-0.47)	0.2 (0.06-0.4)	-2.08 (-2.42--1.73)
Andorra	2.28 (0.8-4.1)	3.95 (1.46-7.21)	0.73	4.19 (1.48-7.55)	4.62 (1.71-8.43)	-1.18 (-1.55--0.81)
Angola	126.71 (53.84-213.86)	90.85 (37.78-159.07)	-0.28	1.23 (0.52-2.08)	0.28 (0.12-0.49)	-5.86 (-6.45--5.27)
Antigua and Barbuda	9.62 (4.23-14.62)	14.87 (6.85-22.75)	0.55	15.98 (7.02-24.28)	16.64 (7.67-25.45)	-0.94 (-1.09--0.79)
Argentina	3094.33 (1429.32-4750.29)	4693.49 (2131.71-7440.98)	0.52	9.35 (4.32-14.35)	10.32 (4.69-16.36)	0.43 (0.12-0.75)
Armenia	145.79 (65.21-223.59)	99.38 (42.7-157.66)	-0.32	4.26 (1.91-6.54)	3.32 (1.43-5.26)	-3.75 (-4.25--3.24)
Australia	2022.27 (903.38-3163.76)	2026.7 (925.94-3351.86)	0	12 (5.36-18.77)	7.86 (3.59-13)	-3.21 (-3.53--2.88)
Austria	624.39 (286.95-958.51)	291.1 (130.57-496.28)	-0.53	8.04 (3.69-12.34)	3.24 (1.45-5.53)	-3.87 (-4.13--3.61)
Azerbaijan	190.53 (88.55-300.51)	97.58 (37.79-163.74)	-0.49	2.6 (1.21-4.1)	0.93 (0.36-1.56)	-6.19 (-6.87--5.51)
Bahrain	1.49 (0.62-2.77)	4.33 (1.48-8.14)	1.91	0.29 (0.12-0.55)	0.28 (0.1-0.53)	-1.99 (-2.18--1.8)
Bangladesh	5993.3 (2703.56-9596.97)	9865.94 (4415.92-16430.2)	0.65	5.49 (2.48-8.79)	5.99 (2.68-9.98)	-1.81 (-1.92--1.7)
Barbados	27.79 (11.91-43.27)	43.64 (17.66-71.82)	0.57	10.97 (4.7-17.07)	14.59 (5.91-24.02)	0.18 (-0.06-0.41)

Belarus	73.71 (29.53-134.3)	136.3 (55.81-255.57)	0.85	0.71 (0.28-1.29)	1.46 (0.6-2.74)	0.23 (-0.66-1.13)
Belgium	1069.32 (481.04-1612.36)	773.25 (346.8-1256.85)	-0.28	10.72 (4.82-16.16)	6.74 (3.02-10.96)	-2.24 (-2.37--2.1)
Belize	1.26 (0.57-1.94)	4.01 (1.77-6.53)	2.18	0.67 (0.3-1.04)	0.94 (0.41-1.52)	0 (-0.32-0.33)
Benin	2.34 (0.92-4.34)	4.59 (1.72-9.18)	0.96	0.05 (0.02-0.09)	0.03 (0.01-0.07)	-0.32 (-0.99-0.34)
Bermuda	3.29 (1.43-5.16)	11.87 (4.98-19.28)	2.61	5.54 (2.41-8.69)	18.67 (7.83-30.35)	2.04 (1.59-2.49)
Bhutan	13.68 (5.74-23.19)	13.55 (5.55-24.14)	-0.01	2.17 (0.91-3.68)	1.79 (0.73-3.19)	-2.84 (-2.96--2.73)
Bolivia	132.05 (54.78-216.99)	376.52 (153.68-652.4)	1.85	2.07 (0.86-3.4)	3.19 (1.3-5.53)	-0.02 (-0.28-0.25)
Bosnia and Herzegovina	18.49 (7.35-32.96)	25.03 (9.32-49.27)	0.35	0.41 (0.16-0.73)	0.76 (0.28-1.49)	-0.38 (-0.85-0.1)
Botswana	10.45 (4.57-17.15)	26.63 (11.16-46.67)	1.55	0.79 (0.35-1.3)	1.11 (0.47-1.95)	-0.51 (-1.06-0.04)
Brazil	3965.9 (1765.3-6037.03)	8718.57 (3950.1-13562.58)	1.2	2.67 (1.19-4.07)	3.96 (1.79-6.15)	-1.09 (-1.3--0.87)
Brunei	10.2 (4.51-16.28)	45.05 (19.6-73.11)	3.42	3.93 (1.74-6.28)	9.99 (4.34-16.2)	2.25 (1.9-2.6)
Bulgaria	806 (362.25-1258.19)	1273.47 (541.61-2112.69)	0.58	9.29 (4.17-14.5)	18.76 (7.98-31.13)	0.66 (-0.06-1.38)
Burkina Faso	13.77 (5.69-23.81)	17.22 (6.35-31.71)	0.25	0.14 (0.06-0.25)	0.08 (0.03-0.14)	-1.96 (-2.27--1.64)
Burundi	1.84 (0.6-3.73)	19.47 (7.06-36.32)	9.58	0.03 (0.01-0.07)	0.15 (0.05-0.27)	4.68 (3.92-5.44)
Cambodia	1639.58 (668.91-2711.52)	3595.57 (1669.22-5905.07)	1.19	15.96 (6.51-26.4)	21.1 (9.79-34.65)	-0.82 (-0.92--0.73)
Cameroon	15.95 (6.56-27.42)	21.75 (7.65-44.61)	0.36	0.15 (0.06-0.26)	0.07 (0.02-0.14)	-3.73 (-4.16--3.29)
Canada	1946.06 (876.02-2964.63)	1393.85 (655.19-2240.43)	-0.28	7.14 (3.21-10.88)	3.72 (1.75-5.98)	-3.24 (-3.52--2.96)
Cape Verde	1.3 (0.62-2.04)	4.21 (1.74-6.7)	2.24	0.37 (0.18-0.58)	0.75 (0.31-1.2)	0.63 (0.02-1.25)

Central African Republic	17.82 (7.43-31.11)	35.62 (14.37-67.71)	1	0.65 (0.27-1.14)	0.65 (0.26-1.23)	-0.07 (-0.44-0.3)
Chad	19.7 (8.83-32.99)	26.61 (10.72-47.96)	0.35	0.33 (0.15-0.55)	0.15 (0.06-0.27)	-1.42 (-1.67--1.16)
Chile	619.97 (276.54-953.18)	884.17 (404.95-1421.2)	0.43	4.67 (2.08-7.17)	4.7 (2.15-7.56)	-1.56 (-1.78--1.34)
China	68081.5 (29789.69-1117 51.1)	48099.59 (18866.72-8476 0.77)	-0.29	5.79 (2.53-9.5)	3.38 (1.33-5.96)	-3.77 (-3.86--3.67)
Colombia	829.94 (368.4-1234.85)	1776.07 (787.67-2831.03)	1.14	2.55 (1.13-3.8)	3.62 (1.61-5.77)	-0.3 (-0.8-0.2)
Comoros	2.58 (0.88-4.15)	8.72 (3.49-14.68)	2.38	0.56 (0.19-0.9)	1.17 (0.47-1.97)	1.52 (1.3-1.74)
Congo	43.46 (17.58-70.78)	135.71 (57.91-221.38)	2.12	1.81 (0.73-2.95)	2.52 (1.07-4.11)	0.13 (-0.06-0.32)
Cook Islands	0.02 (0-0.03)	0.01 (0-0.01)	-0.5	0.08 (0.03-0.17)	0.03 (0.01-0.08)	-4.86 (-5.11--4.61)
Costa Rica	102.52 (45.97-152.46)	444.34 (186.42-695.29)	3.33	3.37 (1.51-5.01)	9.36 (3.93-14.64)	1.46 (1.26-1.66)
Cote d'Ivoire	3.41 (1.2-6.14)	4.33 (1.35-9.49)	0.27	0.03 (0.01-0.05)	0.02 (0-0.03)	-3.22 (-3.47--2.96)
Croatia	581.68 (257.83-882.4)	884.3 (410.26-1415.76)	0.52	11.97 (5.3-18.15)	21.01 (9.75-33.64)	-0.09 (-0.29-0.12)
Cuba	698.02 (317.69-1080.2)	119.78 (45.44-220.27)	-0.83	6.44 (2.93-9.96)	1.06 (0.4-1.95)	-8.94 (-10.34--7.5 2)
Cyprus	56.57 (25.34-89.94)	131.03 (57.41-207.03)	1.32	7.27 (3.26-11.56)	9.65 (4.23-15.25)	-0.16 (-0.38-0.05)
Czech Republic	1445.6 (665.71-2202.92)	1317.78 (560.71-2135.96)	-0.09	14.04 (6.47-21.4)	12.39 (5.27-20.09)	-1.91 (-2.23--1.58)
Democratic People's Republic of Korea	481.88 (204.54-849.93)	1418.36 (605.61-2566.17)	1.94	2.34 (0.99-4.13)	5.37 (2.29-9.72)	1.64 (1.42-1.87)
Democratic Republic of the Congo	81.77 (33.17-143.2)	696.11 (273.22-1319.37)	7.51	0.21 (0.09-0.38)	0.77 (0.3-1.47)	4.25 (3.37-5.14)
Denmark	447.22	406.84	-0.09	8.69 (3.95-13.41)	6.95 (3.1-11.51)	-2

	(203.16-689.67)	(181.65-673.76)				(-2.22--1.78)
Djibouti	23.06 (9.54-37.85)	44.72 (18.45-82.9)	0.94	5.57 (2.3-9.14)	3.55 (1.47-6.59)	-3.51 (-3.84--3.19)
Dominica	0.6 (0.26-0.98)	0.54 (0.23-0.94)	-0.1	0.83 (0.36-1.36)	0.81 (0.34-1.41)	-1.47 (-2--0.94)
Dominican Republic	167.24 (74.03-263.99)	312.13 (141.5-493.5)	0.87	2.34 (1.04-3.69)	2.83 (1.28-4.48)	-1.43 (-1.75--1.1)
Ecuador	227.38 (100.32-348.05)	1332.29 (560.04-2136.91)	4.86	2.28 (1.01-3.49)	7.37 (3.1-11.83)	2.54 (1.94-3.15)
Egypt	80.68 (33.27-139.99)	120.24 (42.85-227.19)	0.49	0.15 (0.06-0.25)	0.11 (0.04-0.22)	-0.68 (-1.24--0.11)
El Salvador	12.71 (5.43-20.79)	34.86 (13.87-62.18)	1.74	0.24 (0.1-0.39)	0.54 (0.22-0.96)	1.51 (1.33-1.68)
Equatorial Guinea	6.13 (2.44-10.79)	2.91 (1.05-5.75)	-0.53	1.45 (0.58-2.55)	0.19 (0.07-0.38)	-7.37 (-8.5--6.22)
Eritrea	21.14 (8.08-38.02)	36.81 (14.52-68.09)	0.74	0.62 (0.24-1.12)	0.56 (0.22-1.03)	-0.73 (-0.95--0.51)
Estonia	52.93 (23.61-84.61)	39.47 (17.45-69.94)	-0.25	3.37 (1.51-5.39)	3.01 (1.33-5.34)	-2.98 (-3.35--2.6)
Ethiopia	503.01 (202.23-846.13)	272.14 (112.29-481.77)	-0.46	0.99 (0.4-1.67)	0.25 (0.1-0.44)	-5.57 (-5.93--5.2)
Fiji	7.25 (3.15-11.76)	5.19 (1.88-9.48)	-0.28	0.96 (0.42-1.55)	0.56 (0.2-1.03)	-2.72 (-3.46--1.99)
Finland	348.49 (159.93-542.66)	299.58 (127.36-500.77)	-0.14	6.96 (3.19-10.83)	5.41 (2.3-9.05)	-3.02 (-3.26--2.78)
France	6353.42 (2871.18-9913.77)	6959.53 (3009.9-11233.1)	0.1	11 (4.97-17.16)	10.48 (4.53-16.92)	-1.14 (-1.22--1.05)
Gabon	10.34 (3.8-19)	14.52 (6.04-24.94)	0.4	1.05 (0.39-1.93)	0.8 (0.33-1.37)	-1.08 (-1.18--0.98)
Georgia	90.72 (39.74-146.15)	139.16 (59.13-229.13)	0.53	1.64 (0.72-2.65)	3.86 (1.64-6.35)	1.84 (1.3-2.38)
Germany	6955.1 (3209.91-11207.89)	6361.17 (2786.98-10268.71)	-0.09	8.7 (4.02-14.02)	7.45 (3.26-12.03)	-2.14 (-2.4--1.89)
Ghana	11.42	1 (0.2-2.39)	-0.91	0.08 (0.03-0.13)	0 (0-0.01)	-11.89

	(4.37-20.2)					(-13.31--10.45)
Greece	196.19 (85.82-316.41)	467.11 (202.64-792.95)	1.38	1.89 (0.83-3.05)	4.59 (1.99-7.79)	1.24 (0.97-1.51)
Greenland	4.78 (1.97-7.76)	2.86 (1.11-4.98)	-0.4	8.6 (3.54-13.96)	5.1 (1.99-8.87)	-3.87 (-4.02--3.72)
Grenada	2.88 (1.23-4.52)	4.21 (1.88-6.9)	0.46	3.31 (1.41-5.19)	4.1 (1.83-6.73)	-0.11 (-0.26-0.04)
Guam	0.04 (0.01-0.09)	0.04 (0.01-0.11)	0	0.03 (0.01-0.06)	0.02 (0-0.07)	-2.5 (-2.81--2.19)
Guatemala	8.89 (3.63-15.11)	55.4 (22.35-94.59)	5.23	0.11 (0.04-0.18)	0.35 (0.14-0.6)	1.66 (1.07-2.26)
Guinea	19.19 (8.32-31.04)	35 (13.8-61.54)	0.82	0.32 (0.14-0.52)	0.26 (0.1-0.46)	0.27 (0.01-0.53)
Guinea-Bissau	36.38 (15-57.55)	19.13 (7.5-33.34)	-0.47	3.61 (1.49-5.71)	0.93 (0.36-1.62)	-4.87 (-5.32--4.41)
Guyana	37.78 (16.75-59.63)	28.69 (11.81-46.44)	-0.24	4.85 (2.15-7.65)	3.75 (1.54-6.07)	-1.87 (-2.33--1.41)
Haiti	87.69 (35.21-148.57)	298.36 (126.44-520.24)	2.4	1.37 (0.55-2.33)	2.32 (0.98-4.04)	0.55 (0.13-0.98)
Honduras	9.4 (4.03-15.82)	45.12 (18.12-79.55)	3.8	0.2 (0.09-0.34)	0.45 (0.18-0.79)	1.65 (1.25-2.05)
Hungary	995.53 (432.62-1572.64)	1500.96 (677.88-2381.86)	0.51	9.58 (4.16-15.13)	15.64 (7.06-24.82)	0.02 (-0.47-0.52)
Iceland	29.79 (12.99-46.47)	33.4 (14.58-54.2)	0.12	11.73 (5.12-18.3)	9.53 (4.16-15.47)	-2 (-2.21--1.79)
India	11793.12 (5438.96-18256.37)	17209.84 (7656.84-26287.45)	0.46	1.38 (0.64-2.14)	1.22 (0.54-1.86)	-1.9 (-2.18--1.63)
Indonesia	11851.93 (5202.91-18634.03)	20050.43 (8609.09-33214.18)	0.69	6.41 (2.81-10.07)	7.19 (3.09-11.91)	-1.02 (-1.17--0.87)
Iran	358.85 (160.82-567.9)	515.03 (214.73-825.43)	0.44	0.63 (0.28-0.99)	0.6 (0.25-0.97)	-1.74 (-2.19--1.29)
Iraq	41.32 (17.58-69.02)	367.09 (156.46-620.36)	7.88	0.22 (0.1-0.37)	0.89 (0.38-1.5)	2.98 (2.17-3.81)
Ireland	146.94	154.5	0.05	4.08 (1.8-6.54)	3.13 (1.31-5.3)	-1.41

	(64.99-235.5)	(64.88-261.74)				(-1.66--1.16)
Israel	66.8 (29.25-112.62)	100.07 (42.57-173.67)	0.5	1.35 (0.59-2.27)	1.04 (0.44-1.81)	-1.95 (-2.12--1.78)
Italy	3589.31 (1628.82-5556.81)	4677.88 (2039.56-7191.17)	0.3	6.32 (2.87-9.78)	7.82 (3.41-12.02)	-0.53 (-0.77--0.29)
Jamaica	24.06 (10.61-38.66)	64.78 (25-111.45)	1.69	1.02 (0.45-1.63)	2.31 (0.89-3.98)	1.84 (1.53-2.16)
Japan	6446.1 (2768.13-10156.63)	13423.52 (6034.1-20613.39)	1.08	5.12 (2.2-8.07)	10.51 (4.73-16.14)	0.7 (0.52-0.88)
Jordan	55.27 (24.95-87.38)	164.42 (65.98-276.03)	1.97	1.48 (0.67-2.34)	1.33 (0.54-2.24)	-2.57 (-2.81--2.33)
Kazakhstan	659.51 (270.13-1025.3)	376.24 (173.94-608.96)	-0.43	4.02 (1.65-6.25)	1.98 (0.92-3.21)	-3.75 (-4.58--2.92)
Kenya	49.72 (21.08-81.92)	122.89 (56.06-203.45)	1.47	0.21 (0.09-0.35)	0.25 (0.11-0.41)	-0.15 (-0.28--0.02)
Kiribati	0.46 (0.19-0.76)	0.71 (0.29-1.26)	0.54	0.62 (0.26-1.02)	0.59 (0.24-1.04)	-1.21 (-1.36--1.06)
Kuwait	10.75 (4.74-16.9)	46.23 (20.37-73.81)	3.3	0.63 (0.28-0.98)	0.99 (0.44-1.59)	-0.13 (-0.52-0.25)
Kyrgyzstan	107.42 (46.83-168.02)	64.25 (28.23-103.95)	-0.4	2.41 (1.05-3.76)	0.94 (0.41-1.51)	-4.26 (-4.69--3.83)
Lao People's Democratic Republic	696 (257.08-1156.23)	850.58 (338.61-1435.14)	0.22	16.69 (6.17-27.73)	11.53 (4.59-19.45)	-2.37 (-2.5--2.23)
Latvia	137.05 (60.91-218.92)	131.85 (54.88-210.11)	-0.04	5.16 (2.29-8.24)	7.05 (2.93-11.23)	-1.15 (-1.68--0.63)
Lebanon	10.8 (4.21-19.62)	38.56 (15.39-68.47)	2.57	0.36 (0.14-0.66)	0.7 (0.28-1.24)	2.03 (1.46-2.61)
Lesotho	1.13 (0.39-2.17)	1.58 (0.49-3.41)	0.4	0.07 (0.03-0.14)	0.08 (0.03-0.18)	0.54 (0.37-0.72)
Liberia	21.87 (10.24-35.29)	82.71 (34.13-155.19)	2.78	0.89 (0.42-1.43)	1.51 (0.63-2.84)	1.7 (1.34-2.05)
Libya	26.06 (11.61-42.66)	131.33 (55.77-224.86)	4.04	0.62 (0.28-1.01)	1.91 (0.81-3.27)	1.7 (1.36-2.05)

Lithuania	41.18 (17.18-70.08)	77.65 (33.56-132.97)	0.89	1.12 (0.47-1.91)	2.85 (1.23-4.87)	1.43 (0.88-1.98)
Luxembourg	43.53 (19.83-66.94)	36.02 (15.26-58.15)	-0.17	11.42 (5.2-17.56)	5.59 (2.37-9.03)	-2.89 (-3.14--2.63)
Macedonia	44.98 (20.2-70.12)	70.93 (29.16-120.37)	0.58	2.26 (1.01-3.52)	3.26 (1.34-5.53)	-0.67 (-1.12--0.22)
Madagascar	132.59 (58.71-213.41)	314.83 (127.97-524.72)	1.37	1.11 (0.49-1.79)	1.1 (0.45-1.84)	-0.22 (-0.36--0.08)
Malawi	3.75 (1.4-6.91)	4.91 (1.58-10.32)	0.31	0.04 (0.01-0.07)	0.03 (0.01-0.05)	-2.82 (-3.41--2.24)
Malaysia	1933.34 (895.43-3061.67)	3218.69 (1434.2-5103.19)	0.66	10.94 (5.07-17.33)	10.12 (4.51-16.04)	-1.83 (-2.16--1.5)
Maldives	4.46 (1.81-7.37)	9.88 (4.11-15.84)	1.22	2.01 (0.82-3.32)	1.91 (0.79-3.06)	-2.66 (-3.05--2.27)
Mali	36.36 (16.45-57.32)	25.7 (9.58-45.36)	-0.29	0.42 (0.19-0.66)	0.11 (0.04-0.19)	-3.89 (-4.47--3.3)
Malta	18.77 (8.53-29.61)	18.08 (7.66-28.91)	-0.04	5.06 (2.3-7.99)	4.09 (1.73-6.54)	-1.97 (-2.55--1.39)
Marshall Islands	0.14 (0.06-0.26)	0.23 (0.09-0.45)	0.64	0.31 (0.13-0.57)	0.41 (0.16-0.8)	-0.89 (-0.98--0.8)
Mauritania	14.83 (6.09-23.46)	13.38 (5.42-23.72)	-0.1	0.72 (0.3-1.14)	0.3 (0.12-0.54)	-2.64 (-3.08--2.21)
Mauritius	75.77 (34.61-112.72)	93.66 (39.4-147.11)	0.24	6.91 (3.16-10.29)	7.36 (3.1-11.57)	-2.77 (-3.35--2.19)
Mexico	100.84 (42.76-171.57)	593.85 (254.7-1050.03)	4.89	0.12 (0.05-0.2)	0.46 (0.2-0.81)	2.83 (2.45-3.2)
Micronesia (Federated States of)	0.47 (0.18-0.89)	0.5 (0.19-0.99)	0.06	0.45 (0.17-0.86)	0.49 (0.19-0.97)	-1 (-1.1--0.9)
Moldova	64.36 (26.79-107.96)	132.33 (57.46-231.23)	1.06	1.45 (0.6-2.43)	3.68 (1.6-6.43)	1.58 (1.13-2.03)
Monaco	0.99 (0.4-1.86)	1.02 (0.37-1.88)	0.03	3.26 (1.3-6.11)	2.69 (0.98-4.98)	-0.67 (-0.91--0.42)
Mongolia	168.41 (69.68-268.78)	229.33 (100.25-360.25)	0.36	7.8 (3.23-12.46)	6.87 (3-10.8)	-2.32 (-2.55--2.08

						)
Montenegro	9.26 (4.25-15.01)	14.78 (5.84-24.71)	0.6	1.48 (0.68-2.4)	2.39 (0.94-4)	-0.87 (-1.46--0.27)
Morocco	13.73 (5.13-27.11)	32.37 (10.57-65.63)	1.36	0.05 (0.02-0.11)	0.09 (0.03-0.18)	-0.04 (-0.15-0.08)
Mozambique	45.64 (19.51-71.79)	36.66 (14.59-62.76)	-0.2	0.34 (0.15-0.54)	0.12 (0.05-0.2)	-2.79 (-3.24--2.33)
Myanmar	6817.44 (2790.19-11757. 8)	6122.65 (2657.77-9739.7)	-0.1	16.86 (6.9-29.08)	10.85 (4.71-17.26)	-3.31 (-3.59--3.02)
Namibia	16.37 (7.4-25.91)	33.48 (15.12-57.49)	1.05	1.17 (0.53-1.85)	1.38 (0.62-2.36)	-0.22 (-0.36--0.08)
Nauru	0.01 (0-0.03)	0.02 (0.01-0.05)	1	0.14 (0.04-0.31)	0.21 (0.07-0.42)	0.6 (-0.72-1.94)
Nepal	280.37 (117.57-487.55)	185.05 (77.49-316.23)	-0.34	1.44 (0.6-2.5)	0.59 (0.25-1.02)	-4.21 (-4.85--3.55)
Netherlands	2072.07 (935.01-3147.3)	1776.12 (806.76-2921.31)	-0.14	13.89 (6.27-21.09)	10.32 (4.69-16.97)	-2.66 (-2.97--2.36)
New Zealand	299.59 (136.14-482.76)	380.74 (161.29-618.55)	0.27	8.77 (3.98-14.13)	7.37 (3.12-11.97)	-0.98 (-1.26--0.69)
Nicaragua	24.38 (10.69-38.07)	53.61 (23.32-87.4)	1.2	0.63 (0.27-0.98)	0.8 (0.35-1.31)	-1.83 (-2.15--1.52)
Niger	20.83 (9.36-34.33)	23.88 (9.3-45.73)	0.15	0.26 (0.12-0.43)	0.1 (0.04-0.18)	-3.6 (-4.06--3.13)
Nigeria	192.62 (84.14-330.06)	132.64 (52.24-231.76)	-0.31	0.21 (0.09-0.37)	0.06 (0.02-0.1)	-3.84 (-4.28--3.4)
Niue	0.01 (0-0.01)	0 (0-0.01)	-1	0.3 (0.11-0.6)	0.22 (0.07-0.44)	-2.26 (-2.44--2.08)
Northern Mariana Islands	0.01 (0-0.03)	0.04 (0.01-0.1)	3	0.03 (0.01-0.08)	0.08 (0.02-0.21)	1.74 (1.21-2.28)
Norway	664.26 (302.93-1012.66)	456.99 (205.29-724.34)	-0.31	15.64 (7.13-23.85)	8.43 (3.79-13.37)	-3.28 (-3.51--3.05)
Oman	7.13 (3.11-11.84)	6.69 (2.72-11.53)	-0.06	0.36 (0.16-0.6)	0.14 (0.06-0.25)	-3.37 (-3.92--2.81

						)
Pakistan	2246.34 (1017.97-3535.35)	5322.62 (2345.47-8735.48)	1.37	2.02 (0.92-3.18)	2.26 (1-3.71)	0 (-0.28-0.28)
Palau	0.03 (0.01-0.06)	0.03 (0.01-0.08)	0	0.2 (0.06-0.43)	0.18 (0.05-0.42)	-1.61 (-1.78--1.43)
Palestine	95.71 (43.77-170.65)	242.01 (102.78-392.94)	1.53	4.68 (2.14-8.34)	4.71 (2-7.65)	-0.25 (-0.54-0.04)
Panama	133.37 (59.35-200.94)	397.81 (171.43-634.39)	1.98	5.58 (2.48-8.41)	9.27 (3.99-14.78)	0.24 (-0.02-0.5)
Papua New Guinea	2.69 (0.94-5.17)	5.69 (2.15-11.41)	1.12	0.07 (0.02-0.13)	0.05 (0.02-0.11)	-1.48 (-1.66--1.3)
Paraguay	11.69 (4.84-19.26)	42.04 (16.97-74.45)	2.6	0.29 (0.12-0.48)	0.59 (0.24-1.04)	1.26 (1.03-1.49)
Peru	519.22 (221.73-813.27)	358.74 (157.42-608.15)	-0.31	2.4 (1.02-3.76)	0.99 (0.43-1.68)	-5.19 (-5.82--4.55)
Philippines	4908.04 (2240.59-7541.45)	15192 (6936.14-23871.18)	2.1	7.79 (3.56-11.97)	13.41 (6.12-21.08)	0.65 (0.45-0.84)
Poland	920.44 (410.47-1461.07)	1549.12 (604.11-2561.66)	0.68	2.41 (1.08-3.83)	4.05 (1.58-6.7)	0.09 (-0.29-0.47)
Portugal	420.43 (193.68-673.13)	703.82 (315.2-1132.24)	0.67	4.15 (1.91-6.64)	6.63 (2.97-10.67)	0.35 (0.01-0.69)
Puerto Rico	163.82 (71.72-269.01)	200.69 (85.35-347.97)	0.23	4.53 (1.99-7.45)	6.09 (2.59-10.56)	-1.24 (-1.35--1.13)
Qatar	0.52 (0.18-1.01)	1.73 (0.52-3.35)	2.33	0.12 (0.04-0.23)	0.06 (0.02-0.11)	-3.83 (-4.27--3.4)
Romania	582.62 (253.85-918.01)	574.87 (232.15-971.7)	-0.01	2.49 (1.09-3.93)	3.04 (1.23-5.13)	-1.7 (-2.05--1.34)
Russian Federation	7125.77 (3304.01-10930.51)	9841.4 (4520.31-15307.37)	0.38	4.72 (2.19-7.24)	6.79 (3.12-10.57)	-1.06 (-1.7--0.41)
Rwanda	0.95 (0.27-2.16)	1.94 (0.53-4.66)	1.04	0.01 (0-0.03)	0.01 (0-0.04)	-3.59 (-4.77--2.39)
Saint Kitts and Nevis	8.55 (3.81-12.99)	14.57 (6.26-23.64)	0.7	20.63 (9.19-31.33)	24.85 (10.68-40.31)	0.23 (0.07-0.39)
Saint Lucia	3.12 (1.4-4.81)	13.37 (5.62-21.56)	3.29	2.29 (1.02-3.52)	7.53 (3.17-12.15)	1.7 (1.39-2.01)

Saint Vincent and the Grenadines	3.13 (1.38-4.85)	5.17 (2.31-8.41)	0.65	2.86 (1.26-4.43)	4.53 (2.02-7.37)	-1.27 (-1.6--0.95)
Samoa	0.06 (0.02-0.13)	0.09 (0.03-0.2)	0.5	0.04 (0.01-0.08)	0.04 (0.01-0.09)	-2.71 (-3.47--1.94)
San Marino	1.55 (0.65-2.63)	1.56 (0.61-2.74)	0.01	6.52 (2.73-11.08)	4.77 (1.87-8.36)	-1.55 (-2.01--1.08)
Sao Tome and Principe	0.11 (0.04-0.2)	0.12 (0.04-0.25)	0.09	0.09 (0.03-0.16)	0.06 (0.02-0.12)	-0.81 (-1.6--0.02)
Saudi Arabia	40.77 (17.93-70.24)	169.95 (65.95-300.84)	3.17	0.26 (0.11-0.44)	0.45 (0.17-0.8)	0.91 (0.54-1.29)
Senegal	62.78 (27.43-99.51)	19.48 (7.27-36.83)	-0.69	0.82 (0.36-1.3)	0.12 (0.05-0.23)	-7.29 (-8.28--6.28)
Serbia	534.15 (238.11-841.51)	647.34 (256.44-1071.77)	0.21	5.55 (2.47-8.74)	7.26 (2.88-12.02)	-1.69 (-2.13--1.25)
Seychelles	5.77 (2.6-8.98)	6.11 (2.92-9.91)	0.06	7.92 (3.57-12.32)	5.8 (2.77-9.4)	-1.9 (-2.05--1.75)
Sierra Leone	147.26 (68.61-231.12)	165.59 (66.73-273.66)	0.12	3.55 (1.65-5.57)	1.87 (0.75-3.09)	-1.79 (-1.91--1.67)
Singapore	270.71 (124.86-433.91)	353.03 (150.93-574.61)	0.3	8.88 (4.1-14.24)	6.16 (2.64-10.03)	-3.3 (-3.49--3.11)
Slovakia	554.97 (254.01-861.01)	1081.93 (446.2-1781.1)	0.95	10.5 (4.81-16.3)	19.93 (8.22-32.8)	0.79 (0.18-1.4)
Slovenia	116.85 (49.92-182.63)	116.61 (50.68-193.25)	0	5.92 (2.53-9.25)	5.63 (2.45-9.34)	-2.24 (-2.43--2.04)
Solomon Islands	0.26 (0.07-0.55)	0.77 (0.25-1.59)	1.96	0.08 (0.02-0.16)	0.11 (0.04-0.23)	-0.06 (-0.26-0.15)
Somalia	239.87 (98.64-438.59)	787.04 (334.16-1409.17)	2.28	3.02 (1.24-5.52)	3.64 (1.55-6.52)	0.52 (0.12-0.92)
South Africa	189.84 (84.6-296.81)	643.4 (281.59-1017.85)	2.39	0.51 (0.23-0.8)	1.13 (0.5-1.79)	1.84 (1.55-2.14)
South Korea	2203.01 (963.95-3440.35)	8823.22 (4060.98-14507. 14)	3.01	4.98 (2.18-7.78)	17.11 (7.87-28.13)	0.97 (0.55-1.39)
South Sudan	8.71	14.49	0.66	0.15 (0.05-0.31)	0.15 (0.05-0.29)	-0.2

	(3.17-18.27)	(5.24-28.08)				(-0.37--0.03)
Spain	1892.83 (849.03-2901.23)	4262.49 (1823.14-6743.85)	1.25	4.88 (2.19-7.48)	9.36 (4-14.81)	1.23 (0.92-1.55)
Sri Lanka	148.21 (67.93-226.72)	179.17 (68.46-314.35)	0.21	0.87 (0.4-1.32)	0.8 (0.31-1.41)	-2.11 (-2.47--1.75)
Sudan	318.72 (119.68-568.47)	520.46 (197.53-961.92)	0.63	1.59 (0.6-2.84)	1.2 (0.45-2.22)	-1.22 (-1.27--1.16)
Suriname	41.45 (18.63-62.84)	101.19 (44-160.59)	1.44	10.72 (4.82-16.25)	17.47 (7.6-27.72)	0.11 (-0.17-0.39)
Swaziland	4.33 (1.78-7.47)	16.41 (6.31-29.19)	2.79	0.54 (0.22-0.93)	1.42 (0.55-2.53)	2.55 (1.88-3.23)
Sweden	1146.43 (507.1-1764.05)	721.4 (301.03-1178.41)	-0.37	13.35 (5.9-20.54)	6.95 (2.9-11.36)	-3.17 (-3.35--2.98)
Switzerland	709.06 (308.51-1085.15)	887.42 (397.46-1422.62)	0.25	10.33 (4.49-15.8)	9.95 (4.45-15.94)	-1.15 (-1.53--0.76)
Syria	75.53 (31.56-121.79)	164.35 (68.2-288.85)	1.18	0.59 (0.25-0.96)	1.17 (0.49-2.06)	-0.94 (-1.4--0.47)
Taiwan (Province of China)	1811.91 (814.83-2732.21)	3097.71 (1313.78-4868.94)	0.71	8.89 (4-13.4)	13.11 (5.56-20.6)	-0.62 (-0.84--0.4)
Tajikistan	176.27 (78.05-269.94)	193.13 (80.2-316.78)	0.1	3.28 (1.45-5.03)	1.9 (0.79-3.12)	-3.39 (-3.78--2.99)
Tanzania	60 (24.92-102.65)	116.9 (46.62-206.56)	0.95	0.23 (0.1-0.4)	0.2 (0.08-0.35)	-1.12 (-1.33--0.9)
Thailand	8546.75 (3823.62-13195.56)	17234.58 (7117.51-28834.77)	1.02	15.06 (6.74-23.25)	25.85 (10.67-43.24)	-1.2 (-1.4--1)
The Bahamas	16.87 (7.34-25.39)	51.38 (22.94-81.8)	2.05	6.57 (2.86-9.89)	13.24 (5.91-21.08)	0.66 (0.44-0.89)
The Gambia	7.75 (3.48-11.99)	4.49 (1.88-7.54)	-0.42	0.79 (0.35-1.22)	0.19 (0.08-0.31)	-6.01 (-6.88--5.13)
Timor-Leste	10.72 (4.57-17.75)	26.23 (11.18-42.42)	1.45	1.37 (0.58-2.27)	1.88 (0.8-3.04)	-0.44 (-0.78--0.09)
Togo	2.28 (0.85-4.06)	4.78 (1.53-9.33)	1.1	0.06 (0.02-0.11)	0.06 (0.02-0.11)	-1.22 (-1.72--0.71

						)
Tokelau	0.01 (0-0.01)	0 (0-0.01)	-1	0.5 (0.2-0.91)	0.26 (0.09-0.53)	-3.12 (-3.23--3.01)
Tonga	0.21 (0.08-0.37)	0.19 (0.07-0.36)	-0.1	0.21 (0.08-0.37)	0.18 (0.07-0.34)	-1.36 (-1.45--1.28)
Trinidad and Tobago	58.23 (25.61-88.6)	75.02 (32.16-125.67)	0.29	4.83 (2.13-7.35)	5.38 (2.31-9.02)	-2.9 (-3.31--2.49)
Tunisia	26.88 (10.8-44.94)	45.4 (17.87-86.15)	0.69	0.32 (0.13-0.54)	0.38 (0.15-0.73)	-2.11 (-2.38--1.84)
Turkey	180.7 (70.58-338.89)	318 (121.11-609.3)	0.76	0.31 (0.12-0.59)	0.38 (0.14-0.73)	-0.68 (-0.86--0.49)
Turkmenistan	98.27 (43.86-149.48)	75.62 (34.29-129.19)	-0.23	2.66 (1.19-4.04)	1.47 (0.66-2.5)	-4.07 (-4.53--3.6)
Tuvalu	0.07 (0.03-0.12)	0.05 (0.02-0.1)	-0.29	0.69 (0.28-1.31)	0.43 (0.15-0.83)	-1.74 (-1.94--1.55)
Uganda	20.61 (7.63-37.11)	60.6 (20.67-109.47)	1.94	0.12 (0.04-0.21)	0.14 (0.05-0.25)	0.14 (-0.07-0.35)
Ukraine	1784.89 (794.3-2810.39)	1883.04 (804.58-3286.17)	0.05	3.39 (1.51-5.33)	4.37 (1.87-7.63)	-1.24 (-1.86--0.62)
United Arab Emirates	8.82 (3.2-15.76)	44.57 (17.68-79)	4.05	0.47 (0.17-0.84)	0.46 (0.18-0.82)	1.24 (0.63-1.86)
United Kingdom	9084.22 (3994.41-14330.47)	6365.78 (2893.04-10196.18)	-0.3	15.85 (6.97-25.01)	9.38 (4.26-15.03)	-2.2 (-2.53--1.86)
United States	28250.24 (12888.31-42745.31)	18261.25 (8373.21-28368.23)	-0.35	11.12 (5.07-16.82)	5.49 (2.52-8.53)	-3.12 (-3.32--2.92)
Uruguay	608.65 (272.59-913.87)	344.01 (155.71-550.8)	-0.43	19.39 (8.68-29.11)	10.1 (4.57-16.17)	-3.31 (-3.54--3.07)
Uzbekistan	431.2 (187.92-658.03)	294.43 (124.32-473.96)	-0.32	2.06 (0.9-3.14)	0.86 (0.36-1.38)	-5.37 (-6.02--4.72)
Vanuatu	0.34 (0.12-0.65)	0.52 (0.19-1)	0.53	0.22 (0.08-0.43)	0.16 (0.06-0.32)	-2.64 (-2.95--2.34)
Venezuela	363.12	1422.56	2.92	1.93 (0.84-2.88)	5.34 (2.28-8.99)	0.68

	(157.51-541.74)	(608.09-2394.59)				(0.39-0.98)
Viet Nam	7692.93 (3342.56-11895.11)	18148.8 (8161.69-29470)	1.36	11.28 (4.9-17.43)	18.1 (8.14-29.39)	-0.29 (-0.44--0.14)
Virgin Islands, U.S.	5.88 (2.31-9.48)	4.05 (1.6-7.08)	-0.31	5.54 (2.17-8.94)	4.72 (1.86-8.24)	-2.83 (-3.22--2.43)
Yemen	140.28 (61.26-242.43)	538.94 (232.07-1011.29)	2.84	1.03 (0.45-1.78)	1.6 (0.69-3.01)	0.01 (-0.32-0.34)
Zambia	16.05 (6.56-29.11)	66.49 (22.21-158.2)	3.14	0.2 (0.08-0.37)	0.34 (0.11-0.81)	1.14 (0.61-1.67)
Zimbabwe	41.04 (18.33-67.14)	90.2 (35.34-153.68)	1.2	0.4 (0.18-0.65)	0.58 (0.23-0.99)	0.28 (-0.23-0.79)

Supplementary table 1. The DALYs of TB attributable to high SSB consumption cases and rates in 1990 and 2021 across 204 countries, and the trends from 1990 to 2021.

Location	1990_DALYs cases (95% UI)	2021_DALYs cases (95% UI)	Percentage change	1990_ASMR_per 100000(95% UI)	2021_ASMR_per 100 000(95% UI)	EAPC (95% CI)
Afghanistan	8.53 (2.65-16.46)	31.07 (11.52-59.29)	2.64	0.09 (0.03-0.17)	0.1 (0.04-0.19)	1.79 (0.94-2.65)
Albania	1.68 (0.73-2.63)	1.91 (0.71-3.4)	0.14	0.05 (0.02-0.08)	0.07 (0.03-0.13)	-3.35 (-3.79--2.91)
Algeria	5.8 (2.66-9.01)	5.3 (2.1-9.09)	-0.09	0.02 (0.01-0.04)	0.01 (0-0.02)	-4.29 (-4.75--3.83)
American Samoa	0 (0-0.01)	0 (0-0.01)	NA	0.01 (0-0.01)	0.01 (0-0.01)	-0.96 (-1.22--0.7)
Andorra	0.1 (0.03-0.19)	0.2 (0.07-0.38)	1	0.19 (0.06-0.34)	0.24 (0.09-0.44)	-1.12 (-1.51--0.73)
Angola	4.12 (1.77-6.8)	3.06 (1.32-5.31)	-0.26	0.04 (0.02-0.07)	0.01 (0-0.02)	-5.51 (-6.08--4.93)
Antigua and Barbuda	0.46 (0.2-0.7)	0.67 (0.31-1.03)	0.46	0.76 (0.33-1.16)	0.75 (0.34-1.15)	-0.65 (-0.81--0.48)
Argentina	142 (64.62-215.68)	220.51 (99.32-350.82)	0.55	0.43 (0.2-0.65)	0.48 (0.22-0.77)	0.23 (-0.08-0.53)
Armenia	5.14 (2.31-7.92)	4.68 (2-7.48)	-0.09	0.15 (0.07-0.23)	0.16 (0.07-0.25)	-3.04 (-3.53--2.54)
Australia	88.42 (39.68-138.91)	97.43 (43.14-163.06)	0.1	0.52 (0.24-0.82)	0.38 (0.17-0.63)	-3.25 (-3.56--2.93)
Austria	33.49 (15.57-52.47)	16.87 (7.16-29.49)	-0.5	0.43 (0.2-0.68)	0.19 (0.08-0.33)	-3.88 (-4.12--3.64)
Azerbaijan	6.24 (2.88-9.89)	3.44 (1.35-5.64)	-0.45	0.09 (0.04-0.14)	0.03 (0.01-0.05)	-5.62 (-6.25--4.99)
Bahrain	0.05 (0.02-0.09)	0.14 (0.05-0.26)	1.8	0.01 (0-0.02)	0.01 (0-0.02)	-1.72 (-1.97--1.47)
Bangladesh	204.26 (93.42-327.71)	371.92 (167.7-611.81)	0.82	0.19 (0.09-0.3)	0.23 (0.1-0.37)	-1.62 (-1.79--1.46)
Barbados	1.36 (0.57-2.14)	2.23 (0.91-3.77)	0.64	0.54 (0.23-0.84)	0.74 (0.3-1.26)	0.37 (0.2-0.54)

Belarus	3.12 (1.25-5.68)	6.26 (2.58-11.95)	1.01	0.03 (0.01-0.05)	0.07 (0.03-0.13)	0.44 (-0.42-1.31)
Belgium	58.95 (26.58-90.46)	46.23 (20.06-77.72)	-0.22	0.59 (0.27-0.91)	0.4 (0.17-0.68)	-2.24 (-2.37--2.11)
Belize	0.06 (0.02-0.09)	0.16 (0.07-0.26)	1.67	0.03 (0.01-0.05)	0.04 (0.02-0.06)	-0.08 (-0.42-0.25)
Benin	0.1 (0.04-0.19)	0.18 (0.07-0.36)	0.8	0 (0-0)	0 (0-0)	-0.48 (-1.06-0.1)
Bermuda	0.16 (0.07-0.25)	0.63 (0.27-1.02)	2.94	0.27 (0.12-0.42)	0.99 (0.42-1.61)	1.66 (1.24-2.08)
Bhutan	0.44 (0.19-0.73)	0.53 (0.23-0.95)	0.2	0.07 (0.03-0.12)	0.07 (0.03-0.13)	-2.49 (-2.61--2.38)
Bolivia	5.13 (2.18-8.41)	15.84 (6.61-27.04)	2.09	0.08 (0.03-0.13)	0.13 (0.06-0.23)	0.13 (-0.13-0.39)
Bosnia and Herzegovina	0.75 (0.3-1.31)	1.29 (0.47-2.44)	0.72	0.02 (0.01-0.03)	0.04 (0.01-0.07)	-0.23 (-0.69-0.24)
Botswana	0.38 (0.16-0.61)	0.99 (0.41-1.7)	1.61	0.03 (0.01-0.05)	0.04 (0.02-0.07)	-0.39 (-0.89-0.11)
Brazil	147.62 (65.82-224.15)	336.3 (151.9-526.06)	1.28	0.1 (0.04-0.15)	0.15 (0.07-0.24)	-1.35 (-1.58--1.12)
Brunei	0.36 (0.15-0.57)	1.55 (0.67-2.51)	3.31	0.14 (0.06-0.22)	0.34 (0.15-0.56)	2.14 (1.77-2.51)
Bulgaria	32.65 (14.67-51.2)	62.83 (26.69-103.38)	0.92	0.38 (0.17-0.59)	0.93 (0.39-1.52)	1.03 (0.29-1.78)
Burkina Faso	0.55 (0.22-0.93)	0.69 (0.26-1.27)	0.25	0.01 (0-0.01)	0 (0-0.01)	-1.88 (-2.16--1.59)
Burundi	0.07 (0.02-0.14)	0.67 (0.24-1.24)	8.57	0 (0-0)	0.01 (0-0.01)	4.42 (3.73-5.12)
Cambodia	54.94 (22.66-89.75)	128.98 (59.56-211)	1.35	0.53 (0.22-0.87)	0.76 (0.35-1.24)	-0.57 (-0.66--0.48)
Cameroon	0.61 (0.25-1.03)	0.82 (0.28-1.67)	0.34	0.01 (0-0.01)	0 (0-0.01)	-3.3 (-3.69--2.92)
Canada	93.68	77.48	-0.17	0.34 (0.15-0.54)	0.21 (0.09-0.36)	-3.2

	(41.8-145.92)	(33.42-134.49)				(-3.47--2.93)
Cape Verde	0.07 (0.03-0.11)	0.22 (0.09-0.36)	2.14	0.02 (0.01-0.03)	0.04 (0.02-0.06)	0.96 (0.35-1.58)
Central African Republic	0.57 (0.24-1)	1.1 (0.43-2.05)	0.93	0.02 (0.01-0.04)	0.02 (0.01-0.04)	-0.09 (-0.44-0.27)
Chad	0.81 (0.36-1.36)	1 (0.41-1.74)	0.23	0.01 (0.01-0.02)	0.01 (0-0.01)	-1.3 (-1.55--1.06)
Chile	27.39 (12.41-42.33)	44.24 (20.82-73.8)	0.62	0.21 (0.09-0.32)	0.24 (0.11-0.39)	-1.52 (-1.7--1.33)
China	2100.37 (927.2-3451.54)	1738.8 (693.49-3023.7)	-0.17	0.18 (0.08-0.29)	0.12 (0.05-0.21)	-3.74 (-3.85--3.62)
Colombia	31.87 (14.08-47.85)	74.93 (32.69-121.96)	1.35	0.1 (0.04-0.15)	0.15 (0.07-0.25)	-0.58 (-1.05--0.1)
Comoros	0.09 (0.03-0.14)	0.33 (0.14-0.56)	2.67	0.02 (0.01-0.03)	0.04 (0.02-0.07)	1.76 (1.57-1.95)
Congo	1.49 (0.6-2.42)	4.49 (1.91-7.23)	2.01	0.06 (0.03-0.1)	0.08 (0.04-0.13)	0.18 (0.02-0.35)
Cook Islands	0 (0-0)	0 (0-0)	NA	0 (0-0.01)	0 (0-0)	-4.87 (-5.09--4.66)
Costa Rica	4.31 (1.96-6.46)	18.65 (7.89-29.51)	3.33	0.14 (0.06-0.21)	0.39 (0.17-0.62)	1.24 (1.02-1.46)
Cote d'Ivoire	0.11 (0.04-0.21)	0.15 (0.05-0.31)	0.36	0 (0-0)	0 (0-0)	-2.96 (-3.16--2.77)
Croatia	27.14 (11.92-41.55)	46.82 (21.39-77.73)	0.73	0.56 (0.25-0.85)	1.11 (0.51-1.85)	-0.16 (-0.35-0.04)
Cuba	30.9 (13.79-48.36)	7.23 (2.72-13.31)	-0.77	0.28 (0.13-0.45)	0.06 (0.02-0.12)	-8.08 (-9.28--6.87)
Cyprus	2.83 (1.28-4.47)	6.94 (3.04-10.97)	1.45	0.36 (0.17-0.58)	0.51 (0.22-0.81)	-0.33 (-0.54--0.11)
Czech Republic	68.78 (31.32-103.85)	68.05 (29.55-107.43)	-0.01	0.67 (0.3-1.01)	0.64 (0.28-1.01)	-1.87 (-2.2--1.53)
Democratic People's Republic of Korea	16.25 (7-28.02)	51.53 (21.84-92.08)	2.17	0.08 (0.03-0.14)	0.2 (0.08-0.35)	1.43 (1.2-1.67)
Democratic Republic of the Congo	2.81 (1.16-4.93)	23.74 (9.23-44.39)	7.45	0.01 (0-0.01)	0.03 (0.01-0.05)	4.16 (3.3-5.03)

Denmark	23.55 (10.64-36.76)	24.41 (10.55-40.72)	0.04	0.46 (0.21-0.71)	0.42 (0.18-0.7)	-1.68 (-1.92--1.44)
Djibouti	0.74 (0.31-1.22)	1.52 (0.63-2.75)	1.05	0.18 (0.08-0.3)	0.12 (0.05-0.22)	-3.05 (-3.34--2.76)
Dominica	0.03 (0.01-0.05)	0.03 (0.01-0.05)	0	0.04 (0.02-0.07)	0.04 (0.02-0.07)	-1.37 (-1.81--0.93)
Dominican Republic	6.57 (2.92-10.51)	13.38 (5.89-21.23)	1.04	0.09 (0.04-0.15)	0.12 (0.05-0.19)	-1.49 (-1.85--1.12)
Ecuador	9.77 (4.28-15.15)	55.48 (23.17-88.74)	4.68	0.1 (0.04-0.15)	0.31 (0.13-0.49)	2.28 (1.7-2.87)
Egypt	2.48 (1.04-4.29)	3.96 (1.43-7.59)	0.6	0 (0-0.01)	0 (0-0.01)	-0.26 (-0.81-0.29)
El Salvador	0.5 (0.22-0.82)	1.46 (0.57-2.61)	1.92	0.01 (0-0.02)	0.02 (0.01-0.04)	1.31 (1.14-1.48)
Equatorial Guinea	0.21 (0.08-0.37)	0.1 (0.03-0.19)	-0.52	0.05 (0.02-0.09)	0.01 (0-0.01)	-7.06 (-8.17--5.94)
Eritrea	0.62 (0.24-1.13)	1.17 (0.47-2.17)	0.89	0.02 (0.01-0.03)	0.02 (0.01-0.03)	-0.44 (-0.65--0.22)
Estonia	2.36 (1.06-3.72)	2.27 (0.99-4.01)	-0.04	0.15 (0.07-0.24)	0.17 (0.08-0.31)	-2.43 (-2.76--2.09)
Ethiopia	17.33 (7.22-28.79)	11.37 (4.67-20.08)	-0.34	0.03 (0.01-0.06)	0.01 (0-0.02)	-4.93 (-5.26--4.61)
Fiji	0.23 (0.1-0.38)	0.19 (0.07-0.34)	-0.17	0.03 (0.01-0.05)	0.02 (0.01-0.04)	-2.4 (-3.06--1.73)
Finland	17.58 (8.03-27.9)	18.93 (7.75-32.15)	0.08	0.35 (0.16-0.56)	0.34 (0.14-0.58)	-2.57 (-2.86--2.27)
France	351.42 (163.24-552.45)	421.79 (183.14-690.92)	0.2	0.61 (0.28-0.96)	0.64 (0.28-1.04)	-1.27 (-1.35--1.19)
Gabon	0.4 (0.15-0.73)	0.53 (0.22-0.9)	0.32	0.04 (0.02-0.07)	0.03 (0.01-0.05)	-0.96 (-1.06--0.86)
Georgia	3.38 (1.51-5.5)	6.24 (2.7-10.31)	0.85	0.06 (0.03-0.1)	0.17 (0.07-0.29)	2.38

						(1.87-2.89)
Germany	385.21 (181.47-623.26)	355.98 (150.62-584.05)	-0.08	0.48 (0.23-0.78)	0.42 (0.18-0.68)	-2.5 (-2.75--2.25)
Ghana	0.42 (0.16-0.73)	0.04 (0.01-0.12)	-0.9	0 (0-0)	0 (0-0)	-10.48 (-11.66--9.29)
Greece	10.47 (4.55-17.32)	29.19 (12.6-49.42)	1.79	0.1 (0.04-0.17)	0.29 (0.12-0.49)	1.03 (0.76-1.3)
Greenland	0.16 (0.07-0.27)	0.11 (0.04-0.19)	-0.31	0.29 (0.12-0.48)	0.2 (0.08-0.34)	-3.83 (-3.99--3.67)
Grenada	0.14 (0.06-0.22)	0.18 (0.08-0.3)	0.29	0.16 (0.07-0.26)	0.18 (0.08-0.29)	0.23 (-0.02-0.48)
Guam	0 (0-0)	0 (0-0)	NA	0 (0-0)	0 (0-0)	-3.45 (-3.83--3.08)
Guatemala	0.32 (0.13-0.55)	2.1 (0.86-3.6)	5.56	0 (0-0.01)	0.01 (0.01-0.02)	0.99 (0.36-1.63)
Guinea	0.76 (0.33-1.24)	1.3 (0.54-2.3)	0.71	0.01 (0.01-0.02)	0.01 (0-0.02)	0.16 (-0.06-0.38)
Guinea-Bissau	1.26 (0.52-1.99)	0.64 (0.25-1.1)	-0.49	0.13 (0.05-0.2)	0.03 (0.01-0.05)	-4.2 (-4.62--3.79)
Guyana	1.42 (0.63-2.2)	1.07 (0.44-1.74)	-0.25	0.18 (0.08-0.28)	0.14 (0.06-0.23)	-1.91 (-2.32--1.49)
Haiti	3.2 (1.31-5.44)	10.61 (4.63-18.74)	2.32	0.05 (0.02-0.09)	0.08 (0.04-0.15)	0.43 (0.04-0.82)
Honduras	0.32 (0.13-0.54)	1.73 (0.7-3)	4.41	0.01 (0-0.01)	0.02 (0.01-0.03)	1.86 (1.47-2.25)
Hungary	47.23 (20.19-74)	71.64 (31.38-113.78)	0.52	0.45 (0.19-0.71)	0.75 (0.33-1.19)	-0.27 (-0.76-0.23)
Iceland	1.45 (0.64-2.26)	1.84 (0.8-3)	0.27	0.57 (0.25-0.89)	0.52 (0.23-0.86)	-1.69 (-1.93--1.44)
India	367.67 (169.32-578.97)	605.35 (264.07-924.55)	0.65	0.04 (0.02-0.07)	0.04 (0.02-0.07)	-1.67 (-1.93--1.42)
Indonesia	387.41 (169.36-612.05)	712.48 (310.45-1166.17	0.84	0.21 (0.09-0.33)	0.26 (0.11-0.42)	-0.71 (-0.86--0.5

		)				6)
Iran	11.92 (5.59-19.33)	20.58 (8.65-33.42)	0.73	0.02 (0.01-0.03)	0.02 (0.01-0.04)	-1.49 (-1.92--1.05)
Iraq	1.45 (0.62-2.43)	12.56 (5.38-21.13)	7.66	0.01 (0-0.01)	0.03 (0.01-0.05)	3.09 (2.33-3.85)
Ireland	7.37 (3.35-11.8)	8.29 (3.42-14.27)	0.12	0.2 (0.09-0.33)	0.17 (0.07-0.29)	-1.34 (-1.6--1.07)
Israel	3.47 (1.46-5.97)	5.82 (2.47-10.42)	0.68	0.07 (0.03-0.12)	0.06 (0.03-0.11)	-2.08 (-2.25--1.92)
Italy	179.43 (79.61-278.6)	271.25 (116.23-418.44)	0.51	0.32 (0.14-0.49)	0.45 (0.19-0.7)	-0.47 (-0.74--0.21)
Jamaica	1.26 (0.57-2.12)	3.09 (1.15-5.49)	1.45	0.05 (0.02-0.09)	0.11 (0.04-0.2)	1.57 (1.3-1.85)
Japan	290.69 (122.24-452.91)	824.51 (359.64-1336.77)	1.84	0.23 (0.1-0.36)	0.65 (0.28-1.05)	0.41 (0.26-0.56)
Jordan	1.81 (0.8-2.85)	5.72 (2.39-9.5)	2.16	0.05 (0.02-0.08)	0.05 (0.02-0.08)	-2.3 (-2.5--2.1)
Kazakhstan	23.28 (9.48-36.11)	14.29 (6.67-23.29)	-0.39	0.14 (0.06-0.22)	0.08 (0.04-0.12)	-3.34 (-4.13--2.53)
Kenya	1.72 (0.73-2.83)	4.36 (1.99-7.27)	1.53	0.01 (0-0.01)	0.01 (0-0.01)	0.22 (0.05-0.39)
Kiribati	0.01 (0.01-0.02)	0.02 (0.01-0.04)	1	0.02 (0.01-0.03)	0.02 (0.01-0.03)	-1.11 (-1.25--0.97)
Kuwait	0.35 (0.16-0.56)	1.57 (0.65-2.56)	3.49	0.02 (0.01-0.03)	0.03 (0.01-0.06)	0.05 (-0.39-0.49)
Kyrgyzstan	3.72 (1.64-5.88)	2.19 (0.98-3.49)	-0.41	0.08 (0.04-0.13)	0.03 (0.01-0.05)	-3.89 (-4.34--3.45)
Lao People's Democratic Republic	23.28 (8.7-38)	29.52 (11.87-49.97)	0.27	0.56 (0.21-0.91)	0.4 (0.16-0.68)	-2.06 (-2.18--1.94)
Latvia	6.04 (2.64-9.58)	7.1 (2.93-11.4)	0.18	0.23 (0.1-0.36)	0.38 (0.16-0.61)	-0.74 (-1.29--0.19)
Lebanon	0.46 (0.18-0.83)	2 (0.79-3.55)	3.35	0.02 (0.01-0.03)	0.04 (0.01-0.06)	2.05 (1.47-2.62)
Lesotho	0.05 (0.02-0.09)	0.06 (0.02-0.12)	0.2	0 (0-0.01)	0 (0-0.01)	0.28

						(0.12-0.44)
Liberia	0.88 (0.41-1.42)	2.93 (1.26-5.52)	2.33	0.04 (0.02-0.06)	0.05 (0.02-0.1)	1.64 (1.3-1.98)
Libya	1 (0.45-1.67)	4.81 (2.03-8.03)	3.81	0.02 (0.01-0.04)	0.07 (0.03-0.12)	1.87 (1.55-2.19)
Lithuania	1.88 (0.81-3.25)	4.06 (1.73-6.99)	1.16	0.05 (0.02-0.09)	0.15 (0.06-0.26)	1.48 (0.95-2.02)
Luxembourg	2.21 (1.01-3.4)	2.17 (0.87-3.71)	-0.02	0.58 (0.27-0.89)	0.34 (0.13-0.58)	-2.54 (-2.79--2.28)
Macedonia	1.89 (0.85-2.96)	3.33 (1.38-5.54)	0.76	0.09 (0.04-0.15)	0.15 (0.06-0.25)	-0.37 (-0.9-0.16)
Madagascar	4.59 (2.01-7.45)	10.17 (4.19-16.82)	1.22	0.04 (0.02-0.06)	0.04 (0.01-0.06)	0.01 (-0.13-0.15)
Malawi	0.13 (0.05-0.24)	0.17 (0.06-0.36)	0.31	0 (0-0)	0 (0-0)	-2.68 (-3.22--2.13)
Malaysia	72.03 (33.39-113.39)	129.04 (57.78-202.46)	0.79	0.41 (0.19-0.64)	0.41 (0.18-0.64)	-1.61 (-1.82--1.41)
Maldives	0.15 (0.06-0.24)	0.38 (0.16-0.61)	1.53	0.07 (0.03-0.11)	0.07 (0.03-0.12)	-2.2 (-2.58--1.82)
Mali	1.31 (0.6-2.07)	0.94 (0.34-1.69)	-0.28	0.02 (0.01-0.02)	0 (0-0.01)	-3.71 (-4.26--3.16)
Malta	0.9 (0.4-1.43)	1.06 (0.45-1.78)	0.18	0.24 (0.11-0.39)	0.24 (0.1-0.4)	-1.84 (-2.39--1.3)
Marshall Islands	0 (0-0.01)	0.01 (0-0.01)	Inf	0.01 (0-0.02)	0.01 (0-0.03)	-0.91 (-0.99--0.83)
Mauritania	0.6 (0.25-0.93)	0.57 (0.24-1.02)	-0.05	0.03 (0.01-0.05)	0.01 (0.01-0.02)	-2.38 (-2.8--1.95)
Mauritius	2.75 (1.24-4.08)	3.93 (1.7-6.18)	0.43	0.25 (0.11-0.37)	0.31 (0.13-0.49)	-2.62 (-3.14--2.09)
Mexico	4.15 (1.73-7.28)	22.58 (9.73-39.44)	4.44	0 (0-0.01)	0.02 (0.01-0.03)	2.06 (1.66-2.47)
Micronesia (Federated States of)	0.02 (0.01-0.03)	0.02 (0.01-0.03)	0	0.02 (0.01-0.03)	0.02 (0.01-0.03)	-0.97 (-1.06--0.88)
Moldova	2.5 (1.04-4.2)	5.59 (2.35-9.78)	1.24	0.06 (0.02-0.09)	0.16 (0.07-0.27)	1.47 (1.08-1.86)

Monaco	0.06 (0.02-0.1)	0.06 (0.02-0.11)	0	0.18 (0.07-0.34)	0.16 (0.06-0.28)	-0.61 (-0.85--0.38)
Mongolia	5.8 (2.38-9.2)	7.66 (3.28-12.1)	0.32	0.27 (0.11-0.43)	0.23 (0.1-0.36)	-2.07 (-2.28--1.86)
Montenegro	0.4 (0.19-0.65)	0.75 (0.28-1.26)	0.87	0.06 (0.03-0.1)	0.12 (0.05-0.2)	-0.33 (-0.86-0.21)
Morocco	0.53 (0.2-1.03)	1.27 (0.39-2.57)	1.4	0 (0-0)	0 (0-0.01)	-0.16 (-0.28--0.04)
Mozambique	1.9 (0.82-3.04)	1.46 (0.6-2.48)	-0.23	0.01 (0.01-0.02)	0 (0-0.01)	-2.74 (-3.17--2.31)
Myanmar	227.87 (93.8-384.89)	229.09 (101.52-367.94)	0.01	0.56 (0.23-0.95)	0.41 (0.18-0.65)	-2.95 (-3.21--2.68)
Namibia	0.56 (0.25-0.9)	1.17 (0.52-1.95)	1.09	0.04 (0.02-0.06)	0.05 (0.02-0.08)	-0.13 (-0.25--0.02)
Nauru	0 (0-0)	0 (0-0)	NA	0 (0-0.01)	0.01 (0-0.01)	0.6 (-0.7-1.91)
Nepal	9.07 (3.83-15.46)	6.86 (2.87-11.83)	-0.24	0.05 (0.02-0.08)	0.02 (0.01-0.04)	-3.89 (-4.51--3.25)
Netherlands	103.6 (45.61-158.81)	98.51 (42.97-163.88)	-0.05	0.69 (0.31-1.06)	0.57 (0.25-0.95)	-2.42 (-2.69--2.14)
New Zealand	13.77 (6.02-22.44)	20.63 (8.52-34.45)	0.5	0.4 (0.18-0.66)	0.4 (0.16-0.67)	-0.66 (-0.94--0.38)
Nicaragua	0.89 (0.38-1.37)	2.04 (0.85-3.34)	1.29	0.02 (0.01-0.04)	0.03 (0.01-0.05)	-1.79 (-2.08--1.49)
Niger	0.74 (0.33-1.21)	0.92 (0.37-1.72)	0.24	0.01 (0-0.02)	0 (0-0.01)	-3.2 (-3.63--2.76)
Nigeria	7.72 (3.33-12.91)	5.23 (2.01-9.07)	-0.32	0.01 (0-0.01)	0 (0-0)	-3.57 (-3.97--3.16)
Niue	0 (0-0)	0 (0-0)	NA	0.01 (0.01-0.03)	0.01 (0-0.02)	-2.14 (-2.31--1.97)

Northern Mariana Islands	0 (0-0)	0 (0-0)	NA	0 (0-0)	0 (0-0.01)	1.75 (1.21-2.29)
Norway	35.16 (16.42-54.15)	29.45 (12.79-47.98)	-0.16	0.83 (0.39-1.28)	0.54 (0.24-0.89)	-2.48 (-2.65--2.31)
Oman	0.25 (0.11-0.41)	0.23 (0.09-0.38)	-0.08	0.01 (0.01-0.02)	0 (0-0.01)	-3.02 (-3.66--2.38)
Pakistan	82.31 (37.85-130.26)	181.7 (81.24-293.02)	1.21	0.07 (0.03-0.12)	0.08 (0.03-0.12)	0.09 (-0.17-0.35)
Palau	0 (0-0)	0 (0-0)	NA	0.01 (0-0.02)	0.01 (0-0.02)	-1.36 (-1.54--1.17)
Palestine	3.71 (1.71-6.47)	8.87 (3.91-14.79)	1.39	0.18 (0.08-0.32)	0.17 (0.08-0.29)	-0.13 (-0.46-0.2)
Panama	5.38 (2.36-8.14)	16.81 (7.04-26.5)	2.12	0.23 (0.1-0.34)	0.39 (0.16-0.62)	0.09 (-0.19-0.36)
Papua New Guinea	0.08 (0.03-0.15)	0.17 (0.07-0.33)	1.12	0 (0-0)	0 (0-0)	-1.16 (-1.34--0.98)
Paraguay	0.48 (0.2-0.8)	1.8 (0.71-3.12)	2.75	0.01 (0-0.02)	0.03 (0.01-0.04)	1.36 (1.14-1.58)
Peru	20.91 (9.1-33.21)	17.14 (7.37-30)	-0.18	0.1 (0.04-0.15)	0.05 (0.02-0.08)	-4.87 (-5.44--4.31)
Philippines	157.03 (70.76-240.97)	518.43 (239.16-809.88)	2.3	0.25 (0.11-0.38)	0.46 (0.21-0.72)	0.65 (0.47-0.82)
Poland	41.6 (18.3-66.45)	82.05 (31.68-138.85)	0.97	0.11 (0.05-0.17)	0.21 (0.08-0.36)	0.17 (-0.23-0.56)
Portugal	21.58 (9.76-34.33)	41.54 (18.35-71.06)	0.92	0.21 (0.1-0.34)	0.39 (0.17-0.67)	0.11 (-0.17-0.4)
Puerto Rico	7.17 (3.05-11.78)	10.1 (4.19-17.46)	0.41	0.2 (0.08-0.33)	0.31 (0.13-0.53)	-1.52 (-1.62--1.41)
Qatar	0.01 (0.01-0.03)	0.05 (0.02-0.09)	4	0 (0-0.01)	0 (0-0)	-3.75 (-4.26--3.24)
Romania	22.64 (9.72-36.63)	28 (11.02-48.4)	0.24	0.1 (0.04-0.16)	0.15 (0.06-0.26)	-1.39 (-1.73--1.05)
Russian Federation	287.28	459.3	0.6	0.19 (0.09-0.29)	0.32 (0.15-0.5)	-0.74

	(132.24-440.97)	(214.6-720.88)				(-1.36--0.11)
Rwanda	0.03 (0.01-0.07)	0.07 (0.02-0.18)	1.33	0 (0-0)	0 (0-0)	-2.99 (-4.04--1.93)
Saint Kitts and Nevis	0.39 (0.17-0.6)	0.6 (0.26-0.96)	0.54	0.94 (0.42-1.44)	1.02 (0.44-1.63)	0.52 (0.33-0.71)
Saint Lucia	0.14 (0.06-0.23)	0.61 (0.26-1.01)	3.36	0.11 (0.05-0.17)	0.34 (0.14-0.57)	1.15 (0.9-1.4)
Saint Vincent and the Grenadines	0.14 (0.06-0.22)	0.23 (0.1-0.37)	0.64	0.13 (0.06-0.2)	0.2 (0.09-0.32)	-1.39 (-1.67--1.11)
Samoa	0 (0-0.01)	0 (0-0.01)	NA	0 (0-0)	0 (0-0)	-2.73 (-3.42--2.05)
San Marino	0.08 (0.03-0.14)	0.09 (0.04-0.16)	0.12	0.35 (0.15-0.61)	0.28 (0.12-0.5)	-1.65 (-2.16--1.14)
Sao Tome and Principe	0.01 (0-0.01)	0.01 (0-0.01)	0	0 (0-0.01)	0 (0-0)	-0.55 (-1.22-0.13)
Saudi Arabia	1.35 (0.6-2.29)	4.69 (1.83-7.99)	2.47	0.01 (0-0.01)	0.01 (0-0.02)	0.52 (0.17-0.86)
Senegal	2.41 (1.06-3.78)	0.87 (0.34-1.61)	-0.64	0.03 (0.01-0.05)	0.01 (0-0.01)	-6.4 (-7.3--5.5)
Serbia	22.99 (10.16-35.98)	32.91 (12.7-55.86)	0.43	0.24 (0.11-0.37)	0.37 (0.14-0.63)	-1.67 (-2.06--1.28)
Seychelles	0.24 (0.11-0.38)	0.25 (0.12-0.42)	0.04	0.33 (0.15-0.53)	0.24 (0.12-0.4)	-1.52 (-1.66--1.39)
Sierra Leone	6.01 (2.77-9.34)	6.44 (2.66-10.62)	0.07	0.14 (0.07-0.22)	0.07 (0.03-0.12)	-1.59 (-1.69--1.48)
Singapore	10.42 (4.72-16.83)	17.23 (7.22-28.19)	0.65	0.34 (0.15-0.55)	0.3 (0.13-0.49)	-3.01 (-3.18--2.83)
Slovakia	24.35 (11.1-37.83)	50.99 (20.66-82.42)	1.09	0.46 (0.21-0.72)	0.94 (0.38-1.52)	0.91 (0.33-1.51)
Slovenia	5.52 (2.38-8.79)	6.77 (2.87-11.37)	0.23	0.28 (0.12-0.45)	0.33 (0.14-0.55)	-1.91 (-2.09--1.73)
Solomon Islands	0.01 (0-0.02)	0.02 (0.01-0.05)	1	0 (0-0.01)	0 (0-0.01)	-0.29 (-0.47--0.1)

Somalia	7.37 (3.11-13.29)	24.78 (10.91-44.04)	2.36	0.09 (0.04-0.17)	0.11 (0.05-0.2)	0.69 (0.31-1.07)
South Africa	6.98 (3.1-11.14)	23.87 (10.55-37.61)	2.42	0.02 (0.01-0.03)	0.04 (0.02-0.07)	1.55 (1.29-1.81)
South Korea	81.7 (34.73-128.46)	480.54 (218.04-806.63)	4.88	0.18 (0.08-0.29)	0.93 (0.42-1.56)	1.19 (0.82-1.56)
South Sudan	0.32 (0.11-0.68)	0.5 (0.18-0.96)	0.56	0.01 (0-0.01)	0.01 (0-0.01)	-0.04 (-0.16-0.09)
Spain	97.79 (43.97-154.07)	240.86 (102.73-399.9)	1.46	0.25 (0.11-0.4)	0.53 (0.23-0.88)	1.06 (0.78-1.35)
Sri Lanka	5.51 (2.56-8.39)	7.53 (2.85-12.8)	0.37	0.03 (0.01-0.05)	0.03 (0.01-0.06)	-2.13 (-2.44--1.83)
Sudan	10.77 (4.24-19.76)	17.2 (6.67-31)	0.6	0.05 (0.02-0.1)	0.04 (0.02-0.07)	-1.1 (-1.16--1.04)
Suriname	1.63 (0.72-2.47)	4.09 (1.78-6.66)	1.51	0.42 (0.19-0.64)	0.71 (0.31-1.15)	0.15 (-0.14-0.43)
Swaziland	0.16 (0.06-0.27)	0.54 (0.21-0.95)	2.38	0.02 (0.01-0.03)	0.05 (0.02-0.08)	2.23 (1.64-2.83)
Sweden	62.37 (28.75-97.01)	46.15 (18.59-77.07)	-0.26	0.73 (0.33-1.13)	0.44 (0.18-0.74)	-2.76 (-2.95--2.57)
Switzerland	36.44 (15.97-56.61)	50.9 (22.41-83.97)	0.4	0.53 (0.23-0.82)	0.57 (0.25-0.94)	-0.96 (-1.29--0.62)
Syria	2.52 (1.04-4.02)	6.28 (2.58-10.72)	1.49	0.02 (0.01-0.03)	0.04 (0.02-0.08)	-0.43 (-0.82--0.04)
Taiwan (Province of China)	63.67 (28.85-97.69)	151.83 (65.3-239.05)	1.38	0.31 (0.14-0.48)	0.64 (0.28-1.01)	-0.23 (-0.44--0.02)
Tajikistan	5.93 (2.64-9.1)	6.14 (2.6-10.07)	0.04	0.11 (0.05-0.17)	0.06 (0.03-0.1)	-3.08 (-3.49--2.66)
Tanzania	2.17 (0.9-3.75)	4.4 (1.81-7.8)	1.03	0.01 (0-0.01)	0.01 (0-0.01)	-0.87 (-1.1--0.63)
Thailand	306.29 (138.57-481.1)	703.95 (296.11-1186.27)	1.3	0.54 (0.24-0.85)	1.06 (0.44-1.78)	-1.36 (-1.56--1.17)
The Bahamas	0.61 (0.26-0.91)	2 (0.93-3.21)	2.28	0.24 (0.1-0.36)	0.52 (0.24-0.83)	0.83 (0.58-1.09)

The Gambia	0.28 (0.12-0.43)	0.18 (0.07-0.29)	-0.36	0.03 (0.01-0.04)	0.01 (0-0.01)	-5.47 (-6.25--4.68)
Timor-Leste	0.34 (0.14-0.55)	1.02 (0.43-1.65)	2	0.04 (0.02-0.07)	0.07 (0.03-0.12)	-0.32 (-0.63--0.02)
Togo	0.08 (0.03-0.15)	0.17 (0.06-0.34)	1.12	0 (0-0)	0 (0-0)	-1.15 (-1.61--0.69)
Tokelau	0 (0-0)	0 (0-0)	NA	0.02 (0.01-0.04)	0.01 (0-0.02)	-3.06 (-3.17--2.95)
Tonga	0.01 (0-0.01)	0.01 (0-0.02)	0	0.01 (0-0.01)	0.01 (0-0.02)	-1.18 (-1.27--1.09)
Trinidad and Tobago	2.48 (1.06-3.83)	3.24 (1.4-5.33)	0.31	0.21 (0.09-0.32)	0.23 (0.1-0.38)	-3.03 (-3.39--2.68)
Tunisia	1.08 (0.44-1.81)	1.98 (0.74-3.66)	0.83	0.01 (0.01-0.02)	0.02 (0.01-0.03)	-2.11 (-2.35--1.88)
Turkey	6.61 (2.57-12.12)	14.32 (5.29-27.46)	1.17	0.01 (0-0.02)	0.02 (0.01-0.03)	-0.25 (-0.44--0.06)
Turkmenistan	3.21 (1.43-4.92)	2.46 (1.1-4.21)	-0.23	0.09 (0.04-0.13)	0.05 (0.02-0.08)	-4.05 (-4.49--3.6)
Tuvalu	0 (0-0)	0 (0-0)	NA	0.02 (0.01-0.04)	0.02 (0.01-0.03)	-1.66 (-1.84--1.47)
Uganda	0.78 (0.29-1.4)	2.11 (0.73-3.77)	1.71	0 (0-0.01)	0 (0-0.01)	0.06 (-0.12-0.24)
Ukraine	72.2 (31.55-114.02)	82.34 (34.85-142.84)	0.14	0.14 (0.06-0.22)	0.19 (0.08-0.33)	-1.05 (-1.64--0.45)
United Arab Emirates	0.25 (0.09-0.44)	1.24 (0.47-2.2)	3.96	0.01 (0-0.02)	0.01 (0-0.02)	2.31 (1.58-3.05)
United Kingdom	462.76 (203.96-750.44)	359.05 (164.03-577.47)	-0.22	0.81 (0.36-1.31)	0.53 (0.24-0.85)	-2.07 (-2.4--1.75)
United States	1440.35 (662.8-2215.74)	913.9 (406.96-1442.85)	-0.37	0.57 (0.26-0.87)	0.27 (0.12-0.43)	-3.46 (-3.65--3.27)
Uruguay	29.07 (13.18-44)	19.63 (8.45-31.98)	-0.32	0.93 (0.42-1.4)	0.58 (0.25-0.94)	-3.09 (-3.29--2.9)

Uzbekistan	14.18 (6.14-21.87)	9.54 (4.01-15.54)	-0.33	0.07 (0.03-0.1)	0.03 (0.01-0.05)	-4.99 (-5.57--4.41)
Vanuatu	0.01 (0-0.02)	0.02 (0.01-0.03)	1	0.01 (0-0.01)	0.01 (0-0.01)	-2.39 (-2.64--2.14)
Venezuela	13.66 (5.94-20.31)	56.16 (24.53-94.36)	3.11	0.07 (0.03-0.11)	0.21 (0.09-0.35)	0.49 (0.19-0.79)
Viet Nam	284.25 (124.76-438.98)	682.91 (306.36-1113.48)	1.4	0.42 (0.18-0.64)	0.68 (0.31-1.11)	-0.14 (-0.31-0.04)
Virgin Islands, U.S.	0.23 (0.09-0.38)	0.2 (0.08-0.35)	-0.13	0.22 (0.08-0.36)	0.23 (0.09-0.4)	-3.03 (-3.41--2.66)
Yemen	4.66 (2.03-7.9)	18.29 (7.86-34.05)	2.92	0.03 (0.01-0.06)	0.05 (0.02-0.1)	0.13 (-0.18-0.45)
Zambia	0.55 (0.22-0.99)	2.18 (0.75-4.79)	2.96	0.01 (0-0.01)	0.01 (0-0.02)	1.24 (0.73-1.76)
Zimbabwe	1.59 (0.7-2.62)	3.01 (1.19-5.15)	0.89	0.02 (0.01-0.03)	0.02 (0.01-0.03)	0.01 (-0.41-0.43)

Supplementary table 2. The death of TB attributable to high SSB consumption cases and rates in 1990 and 2021 across 204 countries, and the trends from 1990 to 2021.
