# Supplementary material for: Global burden and temporal trends of tuberculosis attributable to high sugar-sweetened beverage consumption: insights from the Global Burden of Disease Study 2021
Source: Front Nutr. 2025 Oct 28;12:1638390. doi: 10.3389/fnut.2025.1638390 (PMC12602238; doi:10.3389/fnut.2025.1638390)
Supplement: Supplementary file 2 [file Image_1.pdf]

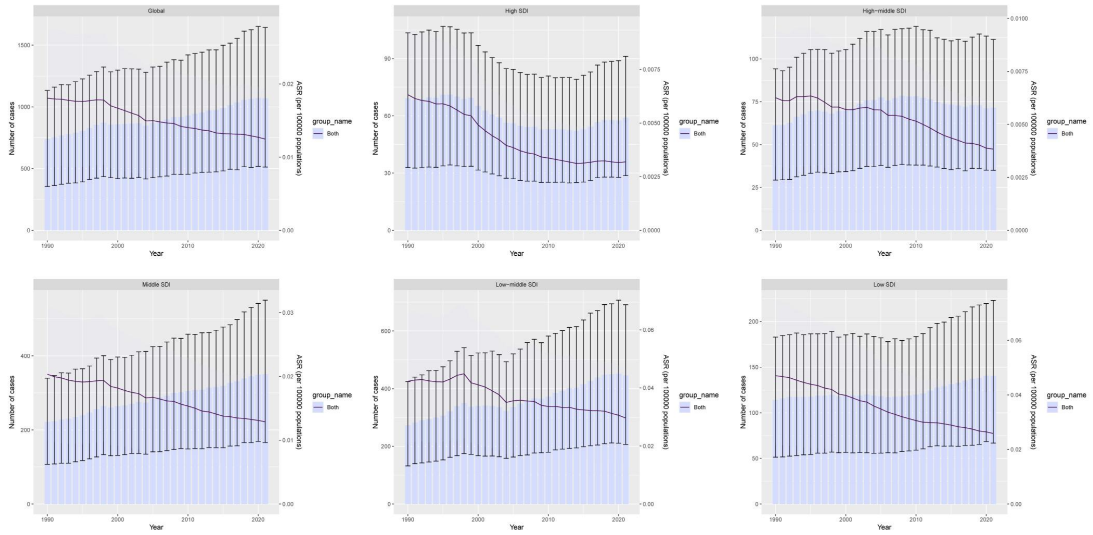

Supplementary figure 1. Death cases and ASMR of TB attributable to high SSB consumption from 1990 to 2021.

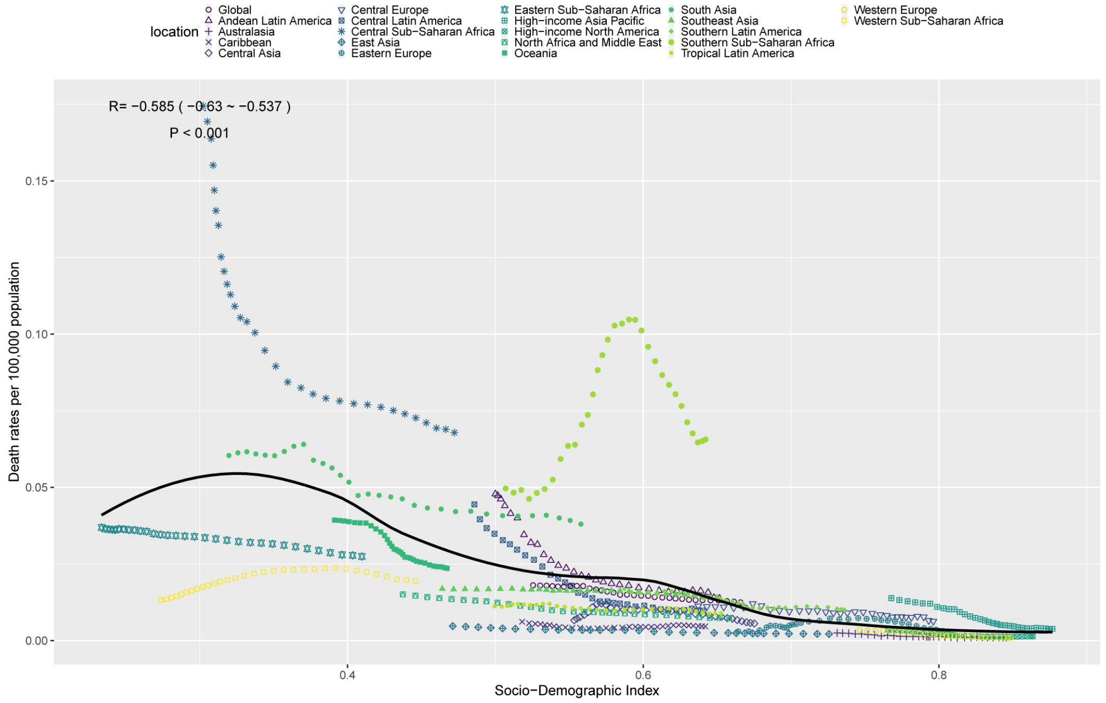

Supplementary figure 2. ASMR of TB attributable to high SSB consumption in 21 GBD regions by SDI, 1990–2021.
